# Etiology, epidemiology, pathology, and advances in diagnosis, vaccine development, and treatment of *Gallibacterium anatis* infection in poultry: a review

**DOI:** 10.1080/01652176.2020.1712495

**Published:** 2020-01-17

**Authors:** Dharanesha Narasinakuppe Krishnegowda, Kuldeep Dhama, Asok Kumar Mariappan, Palanivelu Munuswamy, Mohd. Iqbal Yatoo, Ruchi Tiwari, Kumaragurubaran Karthik, Prakash Bhatt, Maddula Ramakoti Reddy

**Affiliations:** a Division of Pathology, ICAR – Indian Veterinary Research Institute, Bareilly, India; b Sher-E-Kashmir University of Agricultural Sciences and Technology of Kashmir, Srinagar, Jammu and Kashmir, India; c Department of Veterinary Microbiology and Immunology, College of Veterinary Sciences, UP Pandit Deen Dayal Upadhayay Pashu Chikitsa Vigyan Vishwavidyalay Evum Go-Anusandhan Sansthan (DUVASU), Mathura, Uttar Pradesh, India; d Central University Laboratory, Tamil Nadu Veterinary and Animal Sciences University, Chennai, Tamil Nadu, India; e Teaching Veterinary Clinical Complex, College of Veterinary and Animal Sciences, GovindBallabh Pant University of Agriculture and Technology, Pantnagar, Uttarakhand, India

**Keywords:** *Gallibacterium anatis*, poultry, virulence factors, epidemiology, pathogenesis, pathology, diagnosis, treatment, prevention, control, review

## Abstract

*Gallibacterium anatis* is a Gram-negative bacterium of the *Pasteurellaceae* family that resides normally in the respiratory and reproductive tracts in poultry. It is a major cause of oophoritis, salpingitis, and peritonitis, decreases egg production and mortality in hens thereby severely affecting animal welfare and overall productivity by poultry industries across Europe, Asia, America, and Africa. In addition, it has the ability to infect wider host range including domesticated and free-ranging avian hosts as well as mammalian hosts such as cattle, pigs and human. Evaluating the common virulence factors including outer membrane vesicles, fimbriae, capsule, metalloproteases, biofilm formation, hemagglutinin, and determining novel factors such as the RTX–like toxin GtxA, elongation factor-Tu, and clustered regularly interspaced short palindromic repeats (CRISPR) has pathobiological, diagnostic, prophylactic, and therapeutic significance. Treating this bacterial pathogen with traditional antimicrobial drugs is discouraged owing to the emergence of widespread multidrug resistance, whereas the efficacy of preventing this disease by classical vaccines is limited due to its antigenic diversity. It will be necessary to acquire in-depth knowledge on important virulence factors, pathogenesis and, concerns of rising antibiotic resistance, improvised treatment regimes, and novel vaccine candidates to effectively tackle this pathogen. This review substantially describes the etio-epidemiological aspects of *G. anatis* infection in poultry, and updates the recent development in understanding the pathogenesis, organism evolution and therapeutic and prophylactic approaches to counter *G. anatis* infection for safeguarding the welfare and health of poultry.

## 1. Introduction

Diseases in poultry, especially infections caused by resident microbiota, not only severely affect animal welfare, but also are the reason for devastating losses in the poultry industry that are not easily traceable. These microbes result in reduced growth and loss in egg production, thus affecting meat and egg yield in addition to causing mortality. Poultry meat and eggs have become essential dietary components globally, and there is also a huge growing demand for poultry products (AVEC 2011; 2014). The present poultry population worldwide is about 25 billion (FAOSTAT 2016). Currently, about 1500 billion eggs and about 120 million tons of poultry meat are produced annually throughout the globe. Still, the demand for animal products including meat and eggs is increasing along with increases in the global human population and income. Despite enormous growth in the poultry sector, poultry production is still being hampered by numerous factors, with disease being a major one, causing high morbidity and mortality. Undiagnosed diseases cause heavy losses that go unnoticed due to the appearance of general clinical signs and/or pathological changes common to various pathogens (El-Adawy et al. 2018). Septicemia is an important cause of death in poultry, especially chicken, causing substantial economic loss to the poultry industry across the globe. It is well known that several bacterial pathogens such as *Escherichia coli*, *Pasteurella multocida*, *Salmonella* spp., *Staphylococcus aureus, Avibacterium paragallinarum*, *Ornithobacterium rhinotracheale*, and *Klebsiella* spp. are involved in causing septicemia (Fisher et al. 1998; Ewers et al. 2004; Abdul-Aziz et al. 2016). In recent past, several reports of clinical cases in avian and experimental studies in chickens revealed *Gallibacterium anatis* to be an important bacterial pathogen associated with septicemia (Bojesen et al. 2004; Neubauer et al. 2009; Jones et al. 2013; Paudel et al. 2013; Elbestawy 2014; Paudel, Liebhart, Aurich, et al. 2014; Paudel, Liebhart, Hess, et al. 2014; Paudel et al 2015; Persson & Bojesen 2015).

*G. anatis* belongs to the *Pasteurellaceae* family (Christensen, Bisgaard, et al. 2003; Bisgaard et al. 2009) and infects a range of avian host species including chickens, turkeys, ducks, guinea fowls, geese, pheasants, pigeons, peacocks and partridges (Zellner et al. 2004; Rzewuska et al. 2007; Bojesen et al. 2008; Persson & Bojesen 2015; Singh 2016) and has also been reported in non-avian hosts including cattle, horse, pigs, sheep, and rabbits (Kjos-Hansen 1950; Matthes et al. 1969; Janetschke & Risk 1970; Kristensen et al. 2010). Recently, this bacterium has also been isolated from an immunocompromised 26-year-old woman, who developed bacteremia and diarrhea, and in this case it was presumed that the origin of infection was possibly food contaminated by *G. anatis* (Aubin et al. 2013).

In chickens, *G. anatis* has been isolated from clinically healthy birds as part of the normal microbiota in the upper respiratory (nasal and tracheal passages) and lower genital (cloaca and vagina) as well as digestive tracts (rectum) (Bojesen, Nielsen, et al. 2003). Many etiological and epidemiological factors determine the pathogenicity of *G. anatis* in chickens including the bacterial strain, route of infection, and physiological status of host (Bojesen et al. 2008). Host-related factors such as stress, immune status, age, and hormones tend to play a significant role in aggravating disease severity. Co-infection with other pathogenic bacteria or viral agents causing respiratory tract damage, or immunosuppression in the target host, and abrupt change in environmental factors like seasonal variations, cold stress, lack of biosecurity, deficient nutrition, poor ventilation, and overcrowding exacerbate this disease (Gilchrist 1963; Kohlert 1968; Matthes et al. 1969; Bisgaard 1977; Shaw et al. 1990; Mirle et al. 1991; Bojesen et al. 2004; Verbrugghe et al. 2012; Paudel et al. 2015; Persson & Bojesen 2015; Paudel, Hess, et al. 2017; Paudel, Ruhnau, et al. 2017). Coinfection of *G. anatis* with infectious bronchitis virus (IBV) has been reported to increase the rate of systemic infection by *G. anatis* (He-ping et al. 2012;; Mataried 2016), and mixed infection in association with other bacterial pathogens such as *E. coli*, *A. paragallinarum*, and *Mycoplasma gallisepticum* may aggravate disease severity in chickens, resulting in increased morbidity and mortality (Neubauer et al. 2009; Paudel, Hess, et al. 2017; Paudel, Ruhnau, et al. 2017; El-Hamid et al. 2018).

*G. anatis*, particularly the hemolytica biovar, reported to cause oophoritis, salpingitis, peritonitis, perihepatitis, liver necrosis, pericarditis, air sacculitis, tracheitis, enteritis and septicemia in chickens (Bisgaard 1977; Mushin et al. 1980; Shaw et al. 1990; Mirle et al. 1991; Bojesen et al. 2004; Bojesen, Vazquez, Gonzalez, et al. 2007; Neubauer et al. 2009; Paudel et al. 2013). In egg-laying hens, reproductive organs are chiefly affected, and this bacterium produces lesions including hemorrhagic oophoritis and rupture of ovarian follicles (Hacking & Pettit 1974; Jones et al. 1981; Neubauer et al. 2009; Jones et al. 2013; Paudel, Liebhart, Hess, et al. 2014). *G. anatis* has been considered as a primary organism associated with lowered egg production, leading to 8–10% yield reduction and found to cause mortality up to 73% in experimentally immunosuppressed layer chickens (Mirle et al. 1991; Jordan et al. 2005; Neubauer et al. 2009; Shapiro et al. 2013). In cockerels, this bacterium causes epididymitis and leads to decreased semen quality (Paudel, Liebhart, Aurich, et al. 2014). In young chickens the lesions are systemic in nature (Zepeda et al. 2010; Paudel et al. 2013; Zhang et al. 2019).

The wide prevalence of multidrug/antibiotic resistance and considerable antigenic variation observed among *G. anatis* strains are the foremost limitations which lead to treatment failure with the use of antimicrobials and hinder the prevention of this disease by vaccination (Bojesen, Torpdahl, et al 2003; Christensen, Bisgaard, et al. 2003; Bojesen, Bager, et al. 2011; Bojesen, Vazquez, et al. 2011; Johnson et al. 2013; Jones et al. 2013; Chávez et al. 2017; Hess et al.^2019^). Apart from this, other aspects of this pathogen such as virulence factors, pathogenesis, and novel effective vaccine candidates and drugs are yet to be explored in depth by examining recent advances in vaccines and therapeutics. Hence, in this review we discuss the current status of *G. anatis*, with a focus on future prospects. This review details this pathogen’s culture characteristics, different routes of transmission, associated virulence factors, epidemiology, prevalence, host range, pathology, pathogenesis, and advances in diagnosis, treatment, effective prevention and control strategies, and vaccine development for countering this important pathogen’s influence on the poultry industry.

## 2. Etiology

*Gallibacterium* (bacterium of Chicken) is a member of the *Pasteurellaceae* family (Pohl 1981; Christensen, Bisgaard, et al. 2003; Bisgaard et al. 2009). This bacterium was first described as a “hemolytic cloaca bacterium” in 1950 and has been found to be normally present in the cloacae of healthy chickens (Kjos-Hansen 1950). Molecular methods like 16S rRNA sequencing and DNA hybridization suggested that the avian *P. hemolytica* and *A. salpingitidis* complex belong to different genera within *Pasteurellaceae*. Later, an identical bacterium was isolated from several clinically affected poultry and was identified as *Actinobacillus salpingitidis*, or *Pasteurella hemolytica* or *P. anatis* (Harbourne 1962; Gilchrist 1963; Kohlert 1968; Janetschke & Risk 1970; Hacking & Pettit 1974; Bisgaard 1977), before *Gallibacterium* was classified into a separate and independent genus in 2003 (Christensen, Bisgaard, et al. 2003). Recent reclassifications included changing the name of *Pasteurella hemolytica* within the *Pasteurellaceae* family to *Gallibacterium anatis* biovar hemolytica (Christensen, Bisgaard, et al. 2003; Swayne et al. 2013). Currently, the genus consists of seven species, including 4 named species (*Gallibacterium anatis, G. melopsittaci, G. salpingitidis,* and *G. trehalosifermentans*), 3 genomospecies (1, 2, and 3), and unnamed group V (Christensen, Bisgaard, et al. 2003; Bisgaard et al. 2009).

*G. anatis* is a Gram-negative bacterium with pleomorphic cell morphology. It is a facultatively anaerobic, non-spore forming, and non-motile organism (Christensen, Bisgaard, et al. 2003). *G. anatis* has been divided into two phenotypically distinct biovars based on their hemolytic properties: “hemolytica” which causes β-hemolysis, and “anatis” as the non-hemolytic variant. The phenotypic description was done based on Bisgaard (1982) and isolates were characterized subsequently.

### 2.1. Cultural and biochemical characterization of Gallibacterium anatis

*G. anatis* mostly produces a wide β-hemolytic zone with smooth, greyish, non-transparent, shiny colonies on bovine blood agar. These colonies have a butyrous consistency with a margin of 1.0–2.0 mm in diameter within 24–48 hours of incubation at 37°C under aerobic conditions (El-Adawy et al. 2018). They do not produce endospores (Bisgaard 1982). *G. anatis* is a mesophilic and facultatively anaerobic/microaerophilic bacteria positive for catalase, oxidase, and phosphatase tests, and is capable of nitrate reduction (Christensen, Bisgaard, et al. 2003). The *Gallibacterium anatis* biovars are differentiated by analyzing catalase, urease and indole test results, ability to cause hemolysis, ο;-nitrophenyl α-D-glucopyranoside (ONPG) and ρ-nitrophenyl α-D-glucopyranoside (PNPG) tests and acid production without gas formation from fermentation of various sugars, as shown in Table 1 (Christensen, Bisgaard, et al. 2003; Singh 2016; Singh et al. 2016).

**Table 1.** Phenotypic characteristics of *Gallibacterium anatis*.

Character	*G. anatis* biovar haemolytica	*G. anatis* biovar anatis
Hemolysis	+	−
Production of acid from:		
(−) D- Arabinose	(+)	−
(+)L- Arabinose	−	−
Mannitol	+	
m-Inositol	D	D
(−) D- Sorbitol	D	D
(−) L- Fucose	(+)	−
Maltose	D	−
Trehalose	D	+
Dextrin	D	−

Characters are scored as: +, ≥90% of strains positive within 1–2 days; (+), ≥90% of strains positive within 3–14 days; −, <10% of strains positive within 14 days; D, 11–89% of strains positive (Christensen, Foster, et al. 2003; Singh 2016; Singh et al. 2016).

### 2.2. Virulence factors of G. anatis

#### 2.2.1. The RTX–like toxin GtxA

**Figure 1.**
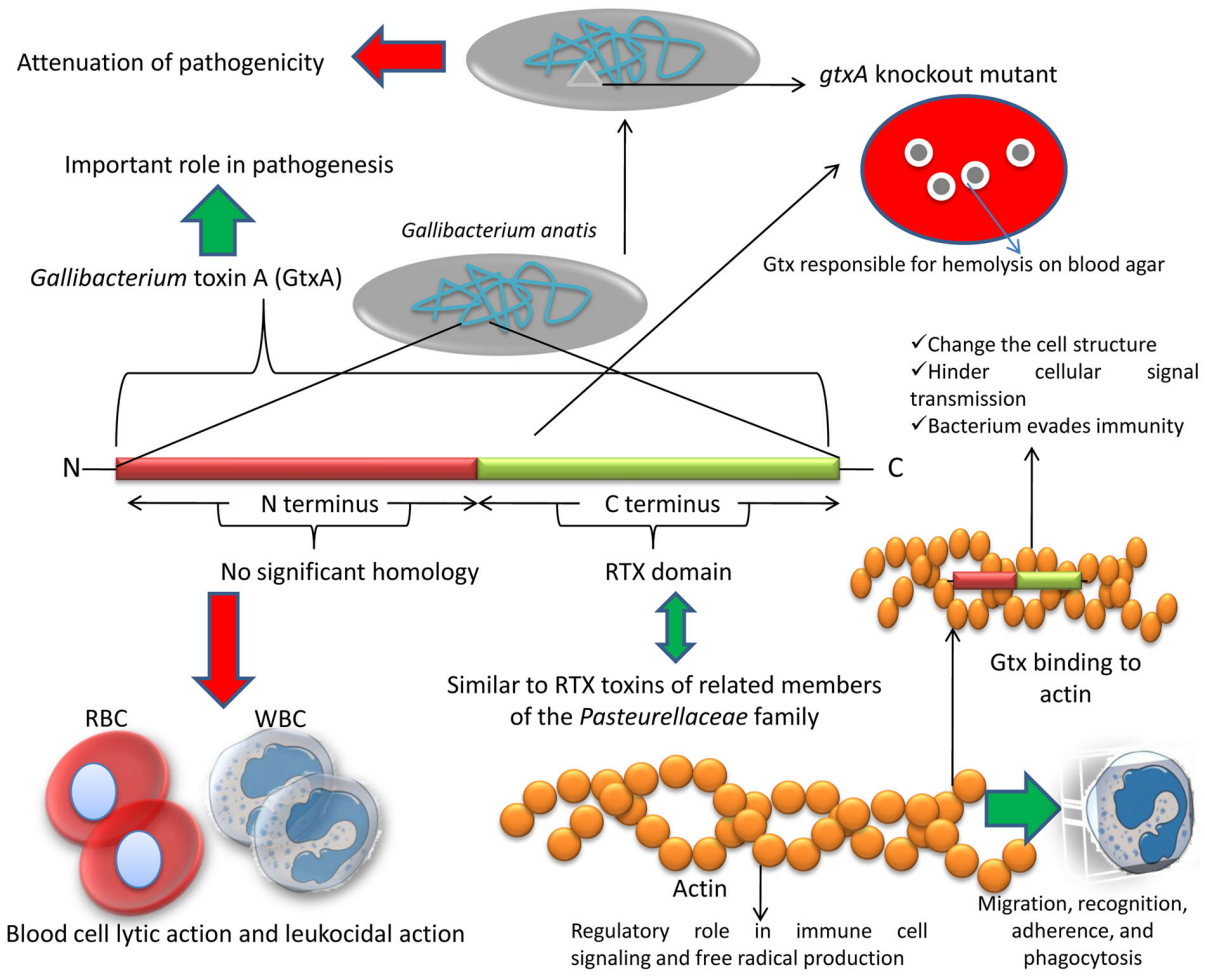
Structure and activity of *Gallibacterium* toxin A. Gtx is a major virulence factor and is involved in hemolytic property of the bacteria.

*Gallibacterium* toxin A (GtxA) is a secreted protein responsible for the hemolytic activity of the *G. anatis* biovar hemolytica, which causes hemolysis around the colony on blood agar, producing a β-hemolytic zone (Kristensen et al. 2010; 2011), and is the most well characterized virulence factor (Figure 1). The GtxA protein causes lysis of red blood cells (RBCs) from a wide variety of hosts and is also leukotoxic, which has been demonstrated experimentally in the chicken macrophage cell line HD11 (Kristensen et al. 2010; Persson & Bojesen 2015). The GtxA protein resembles RTX-toxins of the *Pasteurellaceae* family on sequencing and the α-hemolysin (HlyA) from *E. coli* (Frey & Kuhnert 2002; Shapiro et al. 2013). The GtxA toxin has two domains, the C-terminus and N-terminus. The C-terminus shares similarity with other RTX toxins of related members of the *Pasteurellaceae* family, and the N-terminus has less or no significant matches when compared to sequences in databases. Both the terminal domains are required for maximum hemolytic activities. GtxA may play a role in pathogenesis, as a *gtxA* knockout mutant bacterium exhibited attenuation of pathogenicity (Kristensen et al. 2010; Pors et al. 2014). The N-terminal domain is responsible for blood cell lytic action and leukocidal action. It has weak homology to the Talin protein and helps in protein interactions between the pathogen and host, involving intracellular cytoskeleton proteins such as actin and vaculin (Kristensen et al. 2010). Actin helps cells in migration, recognition, adherence, and phagocytosis. Actin also plays a regulatory role in immune cell signaling and free radical production. Therefore, binding of the GtxA toxin to actin in host immune cells may change the cell structure and hinder cellular signal transmission, and in turn the bacterium can evade the host immune system (Aktories et al. 2011). Besides having hemolytic and leukocytic activity, the exotoxin GtxA induces an immune response (Bager et al. 2014).

#### 2.2.2. Outer membrane vesicles (OMVs)

Outer membrane vesicles (OMVs) are released by the budding of the outer cell wall membrane from Gram-negative bacteria, and hence consist mainly of components of this membrane such as proteins, LPS, and even periplasmic components and DNA (Mashburn**-**Warren & Whiteley 2006; Kulp & Kuehn 2010; MacDonald & Kuehn 2012). *G. anatis* produces OMVs, which are speculated to be involved in adherence, colonization, biofilm formation, or binding and removal of antibacterial substances (Bager, Persson, et al. 2013). *G. anatis* potentially releases hemagglutinin in OMVs, which is capable of agglutinating avian erythrocytes (Zepeda et al. 2009; Ramirez-Apolinar et al. 2012; Bager, Persson, et al. 2013; Johnson et al. 2013; Bager et al. 2014). OMVs from *G. anatis* induce a considerable immune response (Pors, Pedersen, et al. 2016).

#### 2.2.3. Fimbriae

*G. anatis* adheres to epithelial cells as well as other cells in a wide range of hosts, including chickens and other animals. Several pleomorphic fimbriae have been identified and the predominant fimbriae belong to the F17-like family, which are grouped into 1-3 different fimbrial clusters (Kudirkienė et al. 2014; Persson & Bojesen 2015). F17-like fimbriae help bacteria adhere to host mucosal surfaces through binding to N-acetyl-D-glucosamine receptors on host cell surfaces (Klemm & Schembri 2000; Vaca et al. 2011; Lucio et al. 2012; Kudirkienė et al. 2014). The protein adhesin helps in receptor identification and binding (Lintermans et al. 1991; Bouguénec & Bertin 1999). The fimbria protein (FlfA) is very important for virulence *in vivo* and it was demonstrated that an *flfA* knockout mutant (ΔflfA) was considerably attenuated in the natural chicken host without adherence properties (Bager, Nesta, et al. 2013).

#### 2.2.4. Capsule

The bacterial capsule is usually composed of extracellular polysaccharides (Willis & Whitfield 2013). Some strains of *Gallibacterium* have a capsule that contributes to virulence, as in *P. multocida* (Boyce & Adler 2000; Persson & Bojesen 2015). Bojesen, Kristensen, et al. (2011) revealed a thin capsule on *G. anatis* with the help of transmission electron microscopy, but usually the capsule disappears in subcultures (Kjos-Hansen 1950). The capsule on *G. anatis* has been described to play a role in host cell adhesion, cell-cell interaction, and immune evasion (Bojesen, Vazquez, et al. 2011; Singh et al. 2011; Harper et al. 2012).

#### 2.2.5. Metalloproteases

Zinc-containing metalloproteases (MP) help bacteria catalyze peptide bonds in proteins or peptides in addition to helping in colony establishment, nutrition, protection from the host immune system, and bacterial transmission to blood circulation. These enzymes also act on antibodies and complement factors to further down regulate immune function (Miyoshi & Shinoda 2000). The MPs from *G. anatis* are reported to degrade avian IgG, thereby mediating immune evasion (Garcia-Gomez et al. 2005; Chávez et al. 2017).

#### 2.2.6. Biofilm formation

Like many bacteria, *G. anatis* can also form a biofilm, which mainly comprises of proteins, polysaccharides, nucleic acids, and amyloid proteins (40%) (Costerton et al. 1999; Larsen et al. 2007; López-Ochoa et al. 2017). Amyloid proteins are responsible for interacting with various host proteins, such as fibronectin, fibrinogen, laminin and plasminogen and altering the host’s homeostasis (Epstein & Chapman 2008; López-Ochoa et al. 2017). Based on biofilm formation capabilities, *G. anatis* strains can be divided into weak, moderate, and strong groups (Johnson et al. 2013). Biofilm formation is responsible for persistence of infection and disease chronicity, in addition to decreasing sensitivity to antibiotics (Costerton et al. 1999; Donlan & Costerton 2002; Persson & Bojesen 2015).

#### 2.2.7. Hemagglutinin

There are few strains of *G. anatis* that can cause erythrocyte agglutination (Zepeda et al. 2009; Ramirez-Apolinar et al. 2012) and the presence of a potent hemagglutinin in the OMVs was reported (Bager, Persson, et al. 2013; Johnson et al. 2013). Agglutination has been demonstrated in RBCs from broiler chickens, layer hens, quails, rabbits, and pigs using a few subsets of *Gallibacterium* strains. However, most strains agglutinate rabbit erythrocytes. Some *Gallibacterium* strains can agglutinate either avian or mammalian erythrocytes, or both (Zepeda et al. 2009). Recently, a 65-kDa hemagglutinin protein was identified that can bind biotinylated fibrinogen from sheep or pig, and hence can interact with the basement membrane of tissues. This hemagglutinin protein has also been found in biofilms, and therefore may play a role in the pathogenesis of *G. anatis* (Montes-García et al. 2016).

#### 2.2.8. Other putative factors involved in virulence

Elongation factor-Tu (EF-Tu), one of the most abundant proteins in bacterial cells, can cross react with anti-curli polyclonal serum and is also present in biofilms (Furano 1975). This protein is released through vesicle formation (Meneses et al. 2010; Dallo et al. 2012; Bergh et al. 2013). EF-Tu from *G. anatis* possesses amyloid properties, and hence is involved in pathogenesis (López-Ochoa et al. 2017). The *G. anatis* genome also possess clustered regularly interspaced short palindromic repeats (CRISPR), which is considered as bacterial innate defense mechanism and degrades invading foreign nucleic acids (Johnson et al. 2013). Integrative Conjugative Elements (ICEs) have been recognized in the genome of *G. anatis.* ICEs contain antibiotic resistance genes which can excise and become integrated into the genome, which in turn transfers antimicrobial resistance to other bacteria (Wozniak et al. 2009; Johnson et al. 2011; 2013). Strains of *G. anatis* may contain up to four plasmids of different sizes. However, whether these plasmids possess genes encoding antimicrobial resistance and other putative virulence factors is unknown and need further investigation (Christensen, Bisgaard, et al. 2003; Persson & Bojesen 2015). The small colony variants (SCV) are seen in primary cultures of *G. anatis* and have varying hemolytic activity (Greenham & Hill 1962; Harbourne 1962; Janetschke & Risk 1970), and are related to protracted persistence, repeated infections, and emergence of resistance to antimicrobial agents (Proctor et al. 2006). The genes *cps16A* which encodes glycosyltransferase, *cps16B* which encodes HyaE/hyaluronidase, and *cps16F* which encodes UDP-glucose 6-dehydrogenase have been noted to have a role in *G. anatis* virulence (Bossé et al. 2017).

## 3. Transmission

**Figure 2.**
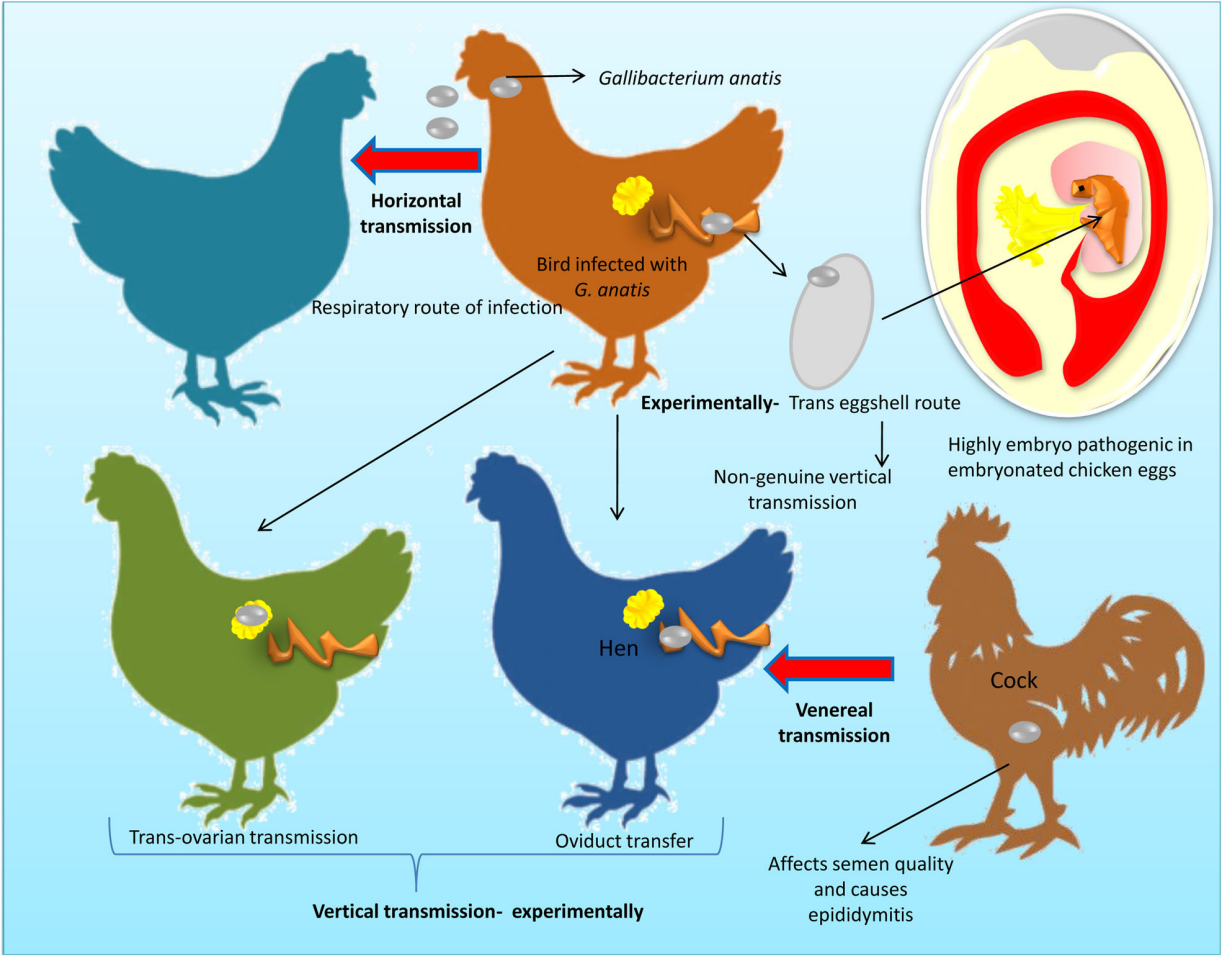
Various modes of transmission of *Gallibacterium anatis*. Horizontal, vertical and venereal transmission was reported. Vertical transmission of *Gallibacterium* was found in experimentally infected hens.

Horizontal dissemination via the respiratory route is the most likely route of transmission and it has been widely accepted (Bisgaard 1977). Another route of entry is through vertical transmission. This phenomenon has recently been demonstrated experimentally in embryonated eggs (Wang, Pors, Olsen, et al. 2018) and occurs either through the trans-ovarian/oviduct route or the trans-eggshell route but there is no direct evidence for natural vertical transmission *in vivo* (Persson & Bojesen 2015). Recently, trans-eggshell penetration and multiplication of *G. anatis* has been documented experimentally and demonstrated to be highly embryo pathogenic in embryonated chicken eggs (Wang, Pors, Olsen, et al. 2018). Different modes of transmission of *G. anatis* are depicted in Figure 2.

*G. anatis* has been noted in four-day-old poultry birds by quantitative PCR (qPCR) (Huangfu et al. 2012) and also been isolated from the egg yolks and ovarian follicles of adult hens ten days after intranasal inoculation, supporting the vertical transmission hypothesis (Kohlert 1968; Janetschke & Risk 1970; Shapiro et al. 2013; Paudel, Liebhart, Hess, et al. 2014). *G. anatis* causes epididymitis and affects semen quality, consistent with the presumed venereal transmission (Paudel, Liebhart, Aurich, et al. 2014). In natural cases, reproductive tract organs are affected mostly through ascending infections (Bojesen, Nielsen, et al. 2003; Neubauer et al. 2009). Septicemia occurs when bacteria, under a favorable environment, gain access to the vascular system in natural habitats (Hacking & Pettit 1974; Shaw et al. 1990; Neubauer et al. 2009; Zepeda et al. 2010; Paudel et al. 2013; Paudel, Liebhart, Hess, et al. 2014).

## 4. Epidemiology, prevalence, and host range

*Gallibacterium* have been reported across the world, such as European countries including Switzerland, Denmark, Germany, Norway, England, Sweden, Czech Republic and Austria (Mráz et al. 1976; Bisgaard 1977; Mirle et al. 1991; Neubauer et al. 2009; Jones et al. 2013); African countries like Nigeria, Egypt, Morocco (Addo & Mohan 1985; Elbestawy et al. 2018; Nassik et al. 2019); Asian countries including China, Taiwan, Iran, Syria, India and Japan (Suzuki et al. 1996; Guo 2011; Huangfu et al. 2012; Singh 2016; Singh et al. 2016; Ataei et al. 2017; Zhang et al. 2017; Singh et al. 2018); American countries such as the USA, Canada, Colombia, Peru and Mexico (Hacking & Pettit 1974; Shaw et al. 1990; Bojesen et al. 2008; Mendoza et al. 2014; Paudel, Liebhart, Aurich, et al. 2014; Paudel, Liebhart, Hess, et al. 2014; Chávez et al. 2017); and Australia (Jordan et al. 2005). *G. anatis*, particularly biovar hemolytica, has recently gained importance due to its ability to cause both respiratory and reproductive infections, and its occurrence in countries on various continents including Europe, Asia, America, and Africa (Neubauer et al. 2009).

*G. anatis* was previously noted as a normal inhabitant of the respiratory tract (Bisgaard 1977) and a common part of the microbiota in the lower genital tract of poultry (Bisgaard 1993; Bojesen, Nielsen, et al. 2003). The *G. anatis* was also reported in normal (Harry 1962; Bisgaard 1977; Mushin et al. 1980; Bojesen, Nielsen, et al. 2003) and affected chickens, and other animals like cattle and pigs (Gerlach 1977; Bisgaard & Dam 1981; Bisgaard 1993; Lin et al. 2001; Christensen, Bisgaard, et al. 2003; Jordan et al. 2005; Bisgaard et al. 2009). *G. anatis* has been isolated from various domestic and non-domestic birds as well (Mushin et al. 1980; Bisgaard 1993; Bojesen, Nielsen, et al. 2003, Rzewuska et al. 2007; Singh 2016; Singh et al. 2016; 2018). Sorour et al. (2015) reported *G. anatis* infection from ducks as well. This bacterium has also been reported in other domestic animals such as horse, sheep, and rabbits (Kjos-Hansen 1950; Matthes et al. 1969; Janetschke & Risk 1970; Kristensen et al. 2010).

## 5. Pathogenesis

*G. anatis* infection produces no specific clinical signs or gross lesions. Based on previous pathological findings and recent investigations where *G. anatis* has been isolated, it was found that *G. anatis* is able to colonize the upper respiratory tract without causing significant clinical signs (Paudel et al. 2013; Paudel, Liebhart, Aurich, et al. 2014; Paudel, Liebhart, Hess, et al. 2014). Several virulence factors have been identified in *G. anatis* as detailed in earlier sections. These bacteria utilize adherence and invasion as their initial virulence mechanisms. Adherence is followed by colonization. Adhesion occurs between bacterial adhesins and receptors on host cells. Once bacteria establish firm adhesion to host cell surfaces, they multiply rampantly and begin synthesizing virulence factors. Previous studies showed that *G. anatis* adheres to chicken oropharyngeal and oviduct epithelial cells during the disease process, and this adhesion has been considered critical for colonizing epithelial surfaces (Vaca et al. 2011; Lucio et al. 2012; Bager, Persson, et al. 2013; Zhang et al. 2017). The highly virulent strains of *G. anatis* have shown higher adherence to primary chicken oviduct epithelial cells (PCOECs) and up regulated production of inflammatory cytokines (IL-6, TNF-α, and IFN-γ), indicating the induction of inflammation and a possible mechanism for inducing tissue injury (Zhang et al. 2017). Many specific virulence factors produced by *G. anatis*, including IgG-degrading proteases, capsule in some strains, hemagglutinin, GtxA, OMVs, fimbriae, metalloproteases, biofilm, EF-Tu, CRISPR, and ICEs, which might influence pathogenicity have been identified (Bojesen et al. 2004; Garcia-Gomez et al. 2005; Christensen et al. 2007; Zepeda et al. 2009; Kristensen et al. 2010; López-Ochoa et al. 2017). Under immunosuppressive conditions, this opportunistic pathogen invades the blood circulation causing septicemia which evokes inflammation in different organs leading to pericarditis, perihepatitis, hepatic necrosis, oophoritis, follicular hemorrhage and rupture, follicular degeneration, salpingitis, peritonitis, enteritis, and inflammation of upper respiratory tract (Harbourne 1962; Gilchrist 1963; Kohlert 1968; Janetschke & Risk 1970; Hacking & Pettit 1974; Bisgaard 1977; Gerlach 1977; Addo & Mohan 1985; Majid et al. 1986; Shaw et al. 1990; Mirle et al. 1991; Suzuki et al. 1996; Neubauer et al. 2009). Mixed infections with other microorganisms such as bacteria, mycoplasma, and viruses (Gilchrist 1963; Matthes et al. 1969; Shaw et al. 1990), hormonal influences (Kohlert 1968; Gerlach 1977), age (Janetschke & Risk 1970; Bisgaard 1977), seasonal changes (Mirle et al. 1991), (cold) stress (Matthes & Löliger 1976; Rzewuska et al. 2007), and immune impairment (Bojesen et al. 2004) are the predisposing factors that enhance the pathogenicity of *G. anatis*. A previous investigation showed that *G. anatis* was the most common single bacterial infection in chickens with reproductive tract disorders (Mirle et al. 1991). Experimental infections with *G. anatis* in cockerels showed decreased semen quality, reduced sperm density, altered total motility, and loss of membrane integrity (Paudel, Liebhart, Aurich, et al. 2014).

## 6. The disease

### 6.1. Clinical manifestations

#### 6.1.1. Clinical signs associated with naturally affected poultry

The clinical signs of *G. anatis* infection are non-specific and multiple systems are affected. Even though *G. anatis* is classified under *Pasteurellaceae*, because of its opportunistic pathogenicity, this species was not assumed to be a major concern in poultry farming. The clinical signs and lesions are nonspecific and confounding, as with other bacterial diseases. *G. anatis* became known as an intriguing agent when it was isolated from clinically affected broiler and breeder flocks. This pathogen has the ability to colonize the upper respiratory tract of broilers and cause no symptoms or mild respiratory signs like rales, coughing, cold, dyspnea, shaking heads, mild swelling of head, and nasal discharge, whereas other signs like diarrhea, anorexia, and general emaciation have also been reported (Bojesen et al. 2008; El-Adawy et al. 2018; Elbestawy et al. 2018). *G. anatis* causes a 3–18% decrease in egg production (Jones et al. 2013). In breeders, *G. anatis* infection is associated with a cumulative mortality rate ranging from 0.06 to 4.9% (Elbestawy et al. 2018). Osuna Chávez et al. (2017) recently collected twenty-three isolates of *G. anatis* from commercial laying hens which had respiratory and reproductive clinical signs in the Sonora region of México. Similarly, in Iran, *G. anatis* was isolated from birds that exhibited reproductive problems (Ataei et al. 2017). In Egypt, *G. anatis* was isolated from 26% of cases of ducks which had respiratory symptoms and systemic infection/septicemic disease. A recent study carried out by El-Hamid et al. (2018) in Egypt found that 23% of chicken flocks which had respiratory and reproductive problems were positive for *G. anatis*.

#### 6.1.2. Clinical signs exhibited in experimentally infected poultry

Upon experimental infection with *G. anatis* in layer hens, whitish diarrhea has been observed from 7 to 24 dpi with a drop in egg production of 66% and 47% after the first and third weeks post-infection, respectively. In later phases, there was gradual rise in egg production, but it was still significantly lower than egg production in the uninfected group (Paudel, Liebhart, Hess, et al. 2014). No clinical signs or gross lesions were noted in experimentally infected *G. anatis* specific pathogen-free (SPF) cockerels. However, the consistency of semen from infected birds was markedly altered in comparison to that from uninfected control groups (Paudel, Liebhart, Aurich, et al. 2014). The clinical signs were found to be exaggerated when *G. anatis* was coinfected with *A. paragallinarum* in SPF white leghorn chickens. The infected birds showed nasal discharge along with infraorbital sinus swelling. The same experiment showed more severe respiratory signs in the coinfection group when compared to symptoms in single-infection and non-vaccinated chickens (Paudel, Hess, et al. 2017). In embryonated eggs, *G. anatis* infects the developing embryo by penetrating through the eggshell and can cause mortality, which is usually higher in *E. coli* co-infected eggs (Wang, Pors, Olsen, et al. 2018). In another experimental infection study in commercial broiler chickens, *G. anatis* infection resulted in depression, respiratory rales, coughing, sneezing, conjunctivitis, teary eyes, reduced feed and water intake, and reduced body weight in the infected group compared to the uninfected group (El-Hamid et al. 2018).

### 6.2. Gross pathology

#### 6.2.1. In naturally affected poultry

*G. anatis* affects the urogenital, gastrointestinal, and respiratory systems in chickens (Ataei et al. 2019). This bacterium has been involved in septicemia, inflammation of ovaries and fallopian tubes, follicle degeneration, and enteric, peritoneal, and respiratory tract infections (Bojesen et al. 2004). Although *G. anatis* is a normal inhabitant of the respiratory and reproductive tract, under favorable conditions it causes reproductive and systemic problems (Neubauer et al. 2009; Jones et al. 2013; Sorour et al. 2015; Ataei et al. 2017; Chávez et al. 2017). The organism could be detected in joints, wattles, lungs, abdomen, heart, and other visceral organs including brain tissue (El-Adawy et al. 2018). A previous report described that amid 141 birds from 31 layer flocks necropsied for reproductive problems, birds from 6 affected flocks were positive for *G. anatis* infection and these suspected birds presented lesions such as peritonitis (21%), hemorrhagic ovaries (18%), ovary regression (40%), hemorrhagic oviducts (12%), nonfunctional oviducts (31%), deformed follicles (28%) and broken follicles (16%). In few birds, fibrinous perihepatitis (3) and pericarditis (2) were also observed (Neubauer et al. 2009).

#### 6.2.2. In experimentally infected poultry

**Figure 3.**
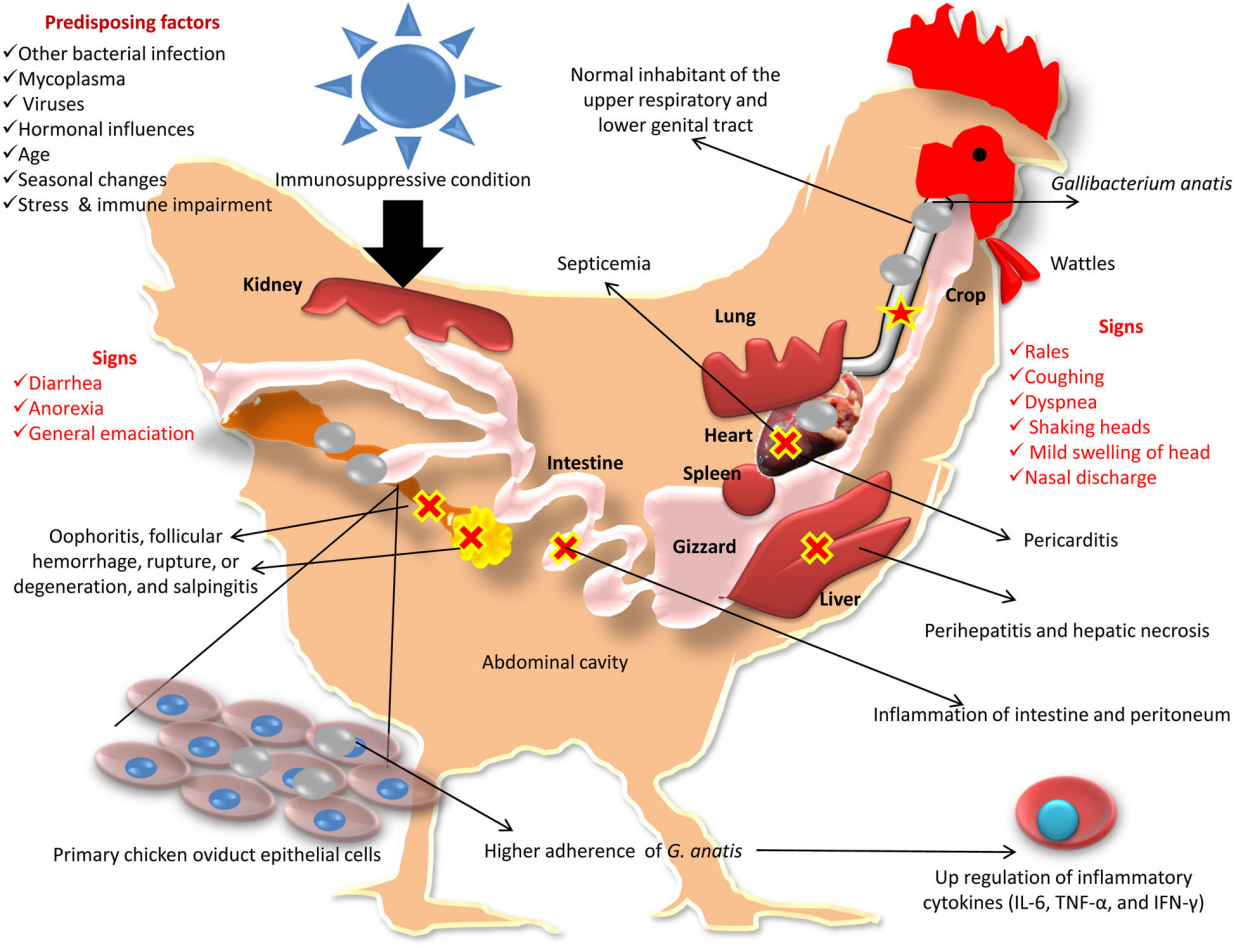
Pathogenesis and clinical signs of *Gallibacterium anatis* infection. 
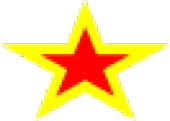
Indicates presence of *G. anatis* in the upper respiratory tract without causing significant clinical signs. 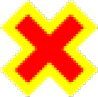
Indicates organs affected and their pathological changes.

A range of gross pathology features has been described in experimental *G. anatis* infections in layer hens, including ruptured follicles, hemorrhagic follicles, pericarditis, multifocal hepatic necrosis, egg degeneration, peritoneal infection, and inflammation and cheese-like deposits in the abdomen (Paudel, Liebhart, Hess, et al. 2014). The severity of gross lesions is reported to vary depending on the strain of *G. anatis*, and has been shown experimentally. Strain 7990 (biovar 3), obtained from clinically affected Mexican chickens, upon experimental infection produced focal or widespread inflammation of the peritoneum, resulting in serous purulent fibrinous fluid exudation, enlarged blood vessels in the ovary, oviduct, and peritoneum and also associated with purulent oophoritis. Further, some experimental studies reported ovary regression and deformation along with exudative focal to diffuse salpingitis in chicken (Paudel et al. 2013; Pors, Skjerning, et al. 2016). The gross lesions such as mild catarrhal tracheitis, congestion of lungs, air sacculitis, pericarditis, accumulation of cheese-like material in the tracheal lumen, ascites, and liver congestion were also reported in experimental infections (El-Hamid et al. 2018). An overview on the pathogenesis and clinical signs of *G. anatis* infection in poultry is depicted in Figure 3.

### 6.3. Histopathology

At present, no reports are available on *G. anatis* produced microscopic lesions in naturally affected chickens, but there are several experimental reports of histopathology induced by this bacterium.

#### 6.3.1. Respiratory organs

Microscopic lesions including necrosis, infiltration, inflammation, and exudation in the respiratory tract were produced upon experimental nasal inoculation of *G. anatis* in chickens, indicating tropism for elements of the respiratory tract such as the trachea, lungs, and air sacs. It has been reported that the most severe lesions have been associated with the *G. anatis* CCM 5976 strain. The lesions consisted of multifocal necrosis with lymphocytic infiltration in the subepithelial lamina of the tracheal epithelium, and moderate inflammation with exudation of fibrin, lymphocytes, and heterophils in the air sac. In the lungs, bronchial goblet cell hyperplasia and hyperplasia of the bronchial lymphoid nodules were reported (Zepeda et al. 2010). In another experimental *G. anatis* or *G. anatis* - *A. paragallinarum* co-infection study in chickens, histopathological findings in the infraorbital sinus and nasal turbinates observed were infiltration of inflammatory cells, predominated by mononuclear cells and heterophils, increased mucosal thickness, and necrosis and sloughing of epithelium (Paudel, Ruhnau, et al. 2017). Few other similar recent experimental *G. anatis* infections in commercial broiler chickens reported the enhanced activity of goblet cells, hyperplasia of the epithelial lining, and inflammatory cell infiltration in tracheal tissue. Severely congested pulmonary blood vessels, hemorrhages, inflammatory cell infiltration, thrombus formation of large blood vessels with perivascular hemorrhage, atrial fusion, fibrinous exudate with RBCs in the lumen, and interstitial pneumonia with granulomatous lesions have been described in lung tissue. In air sacs, focal epithelial hyperplasia, epithelial degeneration, thickening of the membrane due to edema, and inflammatory cellular infiltration were reported (Elbestawy 2014; El-Hamid et al. 2018).

#### 6.3.2. Liver and spleen

Microscopic lesions in the liver have been documented in several experimental infections with selected strains of *G. anatis* in chickens. These lesions include multifocal lymphocytic and heterophilic infiltration amidst multifocal necrosis, hyperplasia of bile ducts, and discrete granulomatous nodules (Bojesen, Christensen, et al. 2003; Bojesen et al. 2004; Zepeda et al. 2010; Paudel et al. 2013; Elbestawy 2014; El-Hamid et al. 2018). In a few other similar experiments, splenic tissues from chickens intravenously inoculated with bacteria showed basophilic bacterial microcolonies along with necrotic splenocytes and eosinophilic material in the ellipsoids (Bojesen, Christensen, et al. 2003; Bojesen et al. 2004; Zepeda et al. 2010; Paudel et al. 2013; Paudel, Liebhart, Hess, et al. 2014).

#### 6.3.3. Reproductive organs

Inflammatory cell infiltration into the follicles is usually noticed in *G. anatis* infection in laying birds. Experimental nasal inoculation of *G. anatis* in layer hens induced lesions in the oviduct including intrafollicular and/or perifollicular infiltration. The lesions were either mild or severe. The infiltrating cells included heterophils and mononuclear cells (Paudel, Liebhart, Hess, et al. 2014). Experimental infection with *G. anatis* (isolate 06-7484-1 TR) in chicks resulted in lymphocytic infiltration in the testes along with degenerative changes in seminiferous tubules on the 28th dpi (Paudel et al. 2013). Similar experiments conducted in SPF cockerels revealed multifocal aggregation of mononuclear cells in interstitial regions of the epididymis at different time intervals (Paudel, Liebhart, Aurich, et al. 2014).

## 7. Diagnosis

Numerous methods are being evaluated for the diagnosis of *G. anatis*, such as phenotypic and cultural characterization, biochemical tests, MALDI-TOF, conventional species-specific PCR, qPCR and also gross and microscopic pathologic examination in infected chickens. The distribution of this organism to various visceral organs can be demonstrated by immunohistochemistry and *in situ* hybridization using specific antibodies and probes, respectively. *G. anatis*-induced inflammation in chickens can be diagnosed by detecting some acute-phase proteins like ovo-transferrin (OTF), and also by quantifying inflammatory mediators. The pro-inflammatory cytokines usually observed are TNF-α, IL-6, and INF-γ (Christensen, Foster, et al. 2003; Bojesen et al. 2008; Alispahic et al. 2011, 2012; Huangfu et al. 2012; Roy et al. 2014; Wang, Pors, Olsen, et al. 2018). Further, other genotypic methods used for strain characterization include DNA–DNA hybridization, pulsed-field gel electrophoresis (PFGE), amplified fragment length polymorphism (AFLP), 16S rRNA analysis, and sequencing genes such as *infB*, *recN*, and *rpoB* (Bisgaard 1977, 1993; Bojesen, Torpdahl, et al. 2003; Christensen, Foster, et al. 2003; Bisgaard et al. 2009).

### 7.1. Hemagglutination assay

*G. anatis* can agglutinate erythrocytes from broiler chickens, layer hens, quails, rabbits, and pigs. Fresh erythrocyte suspensions as well as glutaraldehyde-fixed broiler chicken erythrocytes can be used (Thayer & Beard 1998; Soriano et al. 2002). The hemagglutinating activity can be determined with a suspension of 1% glutaraldehyde-fixed chicken erythrocytes in PBS containing 0.01% thimerosal by the microdilution method/microtiter plates using purified protein or *G. anatis* (Zepeda et al. 2009; Montes-García et al. 2016).

### 7.2. Hemolysis and cytotoxicity assay

The GtxA toxin is mainly responsible for hemolysis and cytotoxicity (Kristensen et al. 2011). A hemolysis assay can be performed using bovine blood. In tris-sodium chloride (TN) buffer (10 mM Tris-HCl, 0.9% NaCl, pH 7.5), the blood is washed repeatedly until the fluid layer become colorless. Then, the washed erythrocytes are incubated with sterile bacterial culture supernatant at a 1:1 ratio at 37 °C for 1 h. The amount of released hemoglobin upon hemolysis is quantified at 540 nm by ELISA (Rowe & Welch 1994; Kristensen et al. 2010). The cytotoxicity assay can be performed by seeding HD11 cells in 96-well plates, adding bacteria or filter-sterilized bacterial culture supernatant, followed by overnight incubation at 37 °C and 5% CO_2_ (Kristensen et al. 2010).

### 7.3. Confocal immunofluorescence microscopy

*G. anatis* culture samples prepared from the mid-logarithmic growth phase (Bager, Nesta, et al. 2013) are fixed with paraformaldehyde on glass slides, blocked with BSA, and incubated with anti-FlfA immune serum. Rhodamine RedX-conjugated goat anti-rabbit secondary antibodies are used for detection. Then, the slides are mounted using suitable mounting agents such as ProLong Gold antifade reagent and images are captured using laser scanning microscopy, and analysis done using suitable software (Bager, Nesta, et al. 2013).

### 7.4. Immunogold electron microscopy

For this method, *G. anatis* is cultured to the mid-logarithmic phase and the obtained cells are processed with PBS. Twenty microliters of *G. anatis* suspension is dispensed on nickel grids coated with Formvar-carbon. The cells are then fixed for 15 min in paraformaldehyde. Anti-FlfA immune serum floats these nickel grids, followed by floating on a secondary antibody that has been conjugated to gold particles. These grids are examined using a transmission electron microscope (Bager, Nesta, et al. 2013).

### 7.5. Serology for ovotransferrin

Acute-phase proteins (APPs) are considered to be good markers. They have important potential for diagnosis and prognosis, as their levels in sera are influenced by inflammation-related events (Olfert et al. 1998). Ovotransferrin (OTF) is considered one of the positive biomarkers among APPs in chicken, and is considered as an imperative diagnostic biomarker for a few selected bacterial infections. OTF can increase 2-fold to ≥ 10-fold during inflammation in chickens, and OTF elevation has been reported in infections caused by *E. coli* in chicken (Xie, Huff, et al. 2002; Xie, Newberry, et al. 2002; Murata et al. 2004). Specific chicken APP concentrations can be quantified efficiently by ELISA (Laursen et al. 1998; Panheleux et al. 2000). Recently, chicken OTF-ELISA has been used for evaluating serum OTF concentrations as an APP in experimental *G. anatis* infections of brown layer chickens (Roy et al. 2014).

### 7.6. Immunohistochemistry (IHC)

Recently, immunochemistry has been used for evaluating the adherence to and invasion of *G. anatis* pathogens in host primary chicken oviduct epithelial cells (PCOECs) using anti-*G. anatis* polyclonal serum raised in rabbits and HRP-labelled goat anti-rabbit antibodies. This IHC assay demonstrated that *G. anatis* pathogens were able to attach epithelial cells without invasion (Zhang et al. 2017).

### 7.7. Fluorescent in situ hybridization (FISH)

This diagnostic tool is very effective in determining the dissemination of *G. anatis* and elucidating its pathogenesis in experimental as well as natural infections. It has been efficiently demonstrated by using a cyanine dye 3.18-labelled *in situ* hybridization probe (GAN850) that targets the 16S-rRNA of *Gallibacterium*. This hybridization technique has been used and evaluated for understanding pathogenic alterations in spleen and liver tissues of experimentally infected chickens (Bojesen, Christensen, et al. 2003, 2004).

### 7.8. MALDI-TOF MS (matrix-assisted laser desorption/ionization time-of-flight mass spectrometry)

MALDI-TOF MS has been a useful application for identifying *G. anatis* (El-Adawy et al. 2018). This technique is used to identify biomarkers based on the size of proteins/peptide molecules by producing fingerprint spectra. These are abundantly present and are products of housekeeping genes having numerous functions. They include ribosomal proteins or DNA- or RNA-binding proteins (Claydon et al. 1996; Suh & Limbach 2004). MALDI-TOF MS requires smaller sample sizes, takes less time for sample analysis, and can process a large number of samples simultaneously, and therefore has great potential for routine laboratory use (Carbonnelle et al. 2011).

The 66 reference strains of *Gallibacterium* were analyzed by MALDI-TOF MS whole-cell fingerprinting and 4 recognized *Gallibacterium* species were rapidly and accurately identified and differentiated with the future possibility of *G. genomospecies* III as a fifth species. Furthermore, one clonal lineage of *G. anatis* has been noted by this approach in many flocks (Alispahic et al. 2011, 2012).

### 7.9. Polymerase chain reaction (PCR)

#### 7.9.1. Conventional Gallibacterium-specific PCR

Many bacterial pathogens, including members of the family *Pasteurellaceae*, may pose differential diagnosis problems based on phenotypic and cultural characterizations. To overcome misdiagnosis, unambiguous genotypic diagnostic tools like PCR have been developed and widely used. An unique 16S to 23S rRNA internal transcribed spacer sequence (ITS) in *Gallibacterium* compared to other members of *Pasteurellaceae* can be used in PCR-based diagnosis (Gürtler & Stanisich 1996; Christensen, Foster, et al. 2003; Bojesen, Vazquez, Robles, et al. 2007). Oligonucleotide primers (Table 2) for 16S rRNA gene was based on 99 sequences (Benson et al. 2004), that represent all *Gallibacterium* and members of *Pasteurellaceae* that are related to it are routinely used for conventional PCR. The primer 1133fgal (5′-TATTCTTTGTTACCARCGG) is specific for *Gallibacterium,* but not other *Pasteurellaceae* members. The 23S rRNA gene sequence primer 114r (5′-GGTTTCCCCATTCGG) has widely been used as a reverse primer (Lane 1991). Further, many investigations have been carried out to identify *Gallibacterium* using internal transcribed spacer (ITS)-PCR), which yields three specific amplicons of approximately 789, 985, and 1032 bp (Neubauer et al. 2009; Singh 2016; Singh et al. 2016; Ataei et al. 2017; Wang, Pors, Olsen, et al. 2018). In many cases, identifying this group of bacteria on a phenotypic basis is difficult, hence, classification based on the gene sequence of the beta-subunit of DNA-dependent RNA-polymerase (*rpoB*) can be useful in those cases (Korczak et al. 2004; Christensen et al. 2007).

**Table 2.** List of primers for PCR of *Gallibacterium anatis*.

Gene	Sequence 5’–3’	References
16S rRNA &23S RNA	TATTCTTTGTTACCARCGG (19)	Bojesen, Vazquez, Robles, et al. 2007
	GGTTTCCCCATTCGG (15)	
*gtxA*	TGCGCAAGTGCTAAATGAAG	Paudel et al. 2013
	GGATAATCGTTGCGCTTTG	
*flfA*	CACCATGGGTGCATTTGCGGATGATCC	Bager, Nesta, et al. 2013
	TATTCGTATGCGATAGTATAGTTC	
*gyrB*	CGATTGTGTCCGTTAAAGTGC	Wang et al. 2018
	TGCAAACGCTCACCAACTG	

PCR can also be performed to detect some of the virulent genes like the GtxA-encoding gene (*gtxA*) and the fimbrial gene (*flfA*) (Sorour et al. 2015). The specific amplification can be done by using primers targeting these sequences. For the *gtx*–N terminus and *flfA*, the primers GalNtxF-TGCGCAAGTGCTAAATGAAG, GalNtxR-GGATAATCGTTGCGCTTTG (Paudel et al. 2013) and 1162F-CACCATGGGTGCATTTGCGGATGATCC, 1162R-TATTCGTATGCGATAGTATAGTTC, have been used, respectively (Bager, Nesta, et al. 2013).

#### 7.9.2. Real-time quantitative PCR (qPCR)

The species-specific identification of *G. anatis* can be done by utilizing real–time quantitative PCR (qPCR) (Huangfu et al. 2012; Wang et al. 2016). This technique can also help quantifying *G. anatis* (Wang et al. 2016; Wang, Pors, Bojesen, et al. 2018). The gyrase subunit B gene (*gyrB*) contains a sequence that is specific to *G. anatis*. It is highly conserved, and is present in prokaryotes in the form of a single-copy gene (Gellert et al. 1976; Stetler et al. 1979; Hsieh & Brutlag 1980; Liu et al. 1980). The protein gyrB is important for the function of DNA gyrase, a DNA replication enzyme, as it encodes the ATPase domain of this enzyme. Thus, *gyrB* is considered a biomarker for diagnostic tests (Huang 1996; Dauga 2002). This gene can be amplified using specific primers (Forward CGATTGTGTCCGTTAAAGTGC, Reverse TGCAAACGCTCACCAACTG) and TaqMan probes (FAM-CCACTACACTTTTCACTTCGG AAGAAACCAG-BHQ) (Wang et al. 2016). This qPCR assay developed by Wang et al. (2016) is assumed to be highly specific, sensitive, and reproducible. It has a detection rate of 97% compared with that of conventional PCR (78%) and culture (34%). Another qPCR developed by Huangfu et al. (2018) that targets *gtxA* gene showed a better detection rate than qPCR based on *gyrB* gene. In general, qPCR needs lower concentration of DNA template and in addition to that it takes less time and is cost effective as compared to conventional PCR and phenotypic identification (Wang et al. 2016).

#### 7.9.3. Loop-mediated isothermal amplification (LAMP) PCR assay

Recently, real-time LAMP was developed and validated for *G. anatis*. This method appeared to be rapid and specific for *G. anatis*. This assay requires 6 sets of primers that amplify the *sodA* gene, a conserved region in *G. anatis*. In LAMP-PCR, isothermal amplification was performed at 63 °C for 60 min, and this method can detect as low as 0.2561 pg of DNA in 34 min. This test has been described as highly sensitive for *G. anatis*. Its high specificity has diagnostic value for *G. anatis* detection. This method is faster and cheaper than quantitative PCR (Stępień-Pyśniak et al. 2018).

### 7.10. Fluorescence-activated cell sorting (FACS) analysis

The bacterial cells which express the fimbriae/antigen can be detected through FACS. *G. anatis* can be harvested from cultures in liquid brain heart infusion (BHI) by centrifugation during the mid-logarithmic growth phase. Then, the bacterial cells are fixed with 1% paraformaldehyde and suspended in a solution of 1% BSA in PBS. Samples with a bacterial cell count of about 2.5 × 10^6^ are poured into the wells of round-bottom 96-well plates. Anti-FlfA immune serum is added to wells and the plate is incubated. Common fluorescein isothiocyanate (FITC)-conjugated goat anti-rabbit secondary antibodies work just like a stain and help in detection. Anti-FlfA preimmune serum can be utilized as a negative control (Bager, Nesta, et al. 2013).

## 8. Therapy and prophylaxis

Although *G. anatis* infection can generally be treated with antimicrobials, some non-responsive cases and recurrence have been reported (Bojesen, Vazquez, et al. 2011; Singh et al. 2016; El-Adawy et al. 2018; Hess et al. 2019). Multi-drug resistant strains of *G. anatis* are frequently reported and these strains show resistance to sulfonamides, novobiocin, tylosin, clindamycin, tetracycline, and penicillin (Jones et al. 2013; Singh 2016; Singh et al. 2016; El-Adawy et al. 2018; Elbestawy et al. 2018). Bojesen, Vazquez, et al. (2011) reported the majority of *G. anatis* strains (65%) to be multidrug resistant in an *in vitro* study and only two strains were found to be susceptible to all antibiotics/drugs tested. Resistance to mainly tetracycline (92%) and sulfamethoxazole (97%) was shown among field strains tested. The genes tet (B), tet (H), and tet (L), which are responsible for tetracycline resistance, have been identified in *G. anatis* (Bojesen, Vazquez, et al. 2011). In India, Singh (2016) reported that *G. anatis* is susceptible to gentamicin, chloramphenicol, azithromycin, nitrofurantoin, ampicillin, imipenem, meropenem, and ertapenem. El-Adawy (2018) reported susceptibility of *G. anatis* to apramycin, florfenicol, and neomycin, but resistance to clindamycin, sulfathiazole, and penicillin. Approximately 93% of field strains tested were reported to be resistant to sulfamethoxine, 93% to spectinomycin, 87% to tylosin, and 80% to oxytetracycline. Since antibiotic susceptibility of strains constantly changes, regular *in vitro* evaluation of the isolates is needed (Elbestawy et al. 2018). A report by Chávez (2017) described marked resistance to penicillin, tylosin, lincomycin, ampicillin, enrofloxacin, oxytetracycline, norfloxacin, and cephalexin; yet sensitive to ceftiofur (73%) and florfenicol (68%). A recent antimicrobial investigation by Hess et al. (2019) reported that 96% isolates from layer flocks had multidrug resistance to different antibiotics, wherein majority of isolates were resistant to tetracycline (89%), tylosin (95%), enrofloxacin (58%), nalidixic acid (77%) and sulfamethoxazole (77%). Various genes have been identified as being responsible for resistance of *G. anatis* to antibiotics. Resistance to tetracycline has been conferred by the tet (31) gene in *G. anatis* (Bojesen, Bager, et al. 2011; Shi et al. 2019). The development of widespread antibiotic resistance (Bojesen, Torpdahl, et al. 2003; Bojesen, Nielsen, et al. 2003; Bojesen, et al. 2011a; Johnson et al. 2013), leads to ineffective treatment. Zeolites, which are aluminosilicate minerals, have been recently found to be effective in reducing about 97% of *G. anatis* microbes in poultry when supplemented in feed (Prasai et al. 2017). In a recent experimental study, the specific chicken egg yolk antibody produced against recombinant N-terminal of GtxA showed considerable protection against disease in *G. anatis*-challenged chickens, resulting in less severe lesions in the peritoneum, liver, and duodenum (Zhang et al. 2019).

Flocks co-infected with other bacterial or viral infections and immunosuppressive agents should be protected by adopting appropriate prophylactic, therapeutic and control measures such as timely vaccination, selective antimicrobial therapy and strict biosecurity measures (He-Ping et al. 2012;; Paudel et al. 2015; Mataried 2016; Paudel, Hess, et al. 2017; Paudel, Ruhnau, et al. 2017; El-Hamid et al. 2018). Any stress that reduces bird resistance to infection must be ameliorated. Trans-eggshell transmission due to fecal contamination of hatching eggs must be prevented by regular cleaning and disinfection of floor and nest material (Paudel, Liebhart, Hess, et al. 2014; Wang, Pors, Olsen, et al. 2018). The evolution of multidrug resistant strains, greater antigenic variation and inefficient clearing of organisms by the infected host are major constrains in preventing the disease.

*G. anatis* possesses many proteins with immunogenic potential and important among these putative vaccine candidates are GtxA, FlfA, Gab_2156, Gab_1309, and Gab_2312. Recombinant proteins such as GtxA-N, GtxA-C, FlfA have been identified as potential vaccine candidates in the recent past (Bager, Nesta, et al. 2013; Bager et al. 2014; Pedersen et al. 2015; Pors, Skjerning, et al. 2016). Recent attempts to generate multivalent and combined vaccines have enabled the prevention of a broad range of diseases using a single vaccination. A killed vaccine (Volvac® Boehringer Ingelheim, Ingelheim, Germany) developed using *G. anatis* and *A. paragallinarum* was reported to protect the immunized birds after experimental challenge (Paudel, Hess, et al. 2017). Laying hens immunized with OMV of *G. anatis* showed decreased lesion severity and higher titers of OMV-specific IgY in serum (Pors et al. 2016b). Chávez et al. (2017) mentioned that the effective immunization against *G. anatis* in chickens is also in practice in Mexico. Administration of OMVs and the fimbrial protein FlfA have been reported to provide considerable protection against *G. anatis* infection, indicating that these antigens could serve as potential future vaccine candidates against *G. anatis* (Persson et al. 2018).

**Figure 4.**
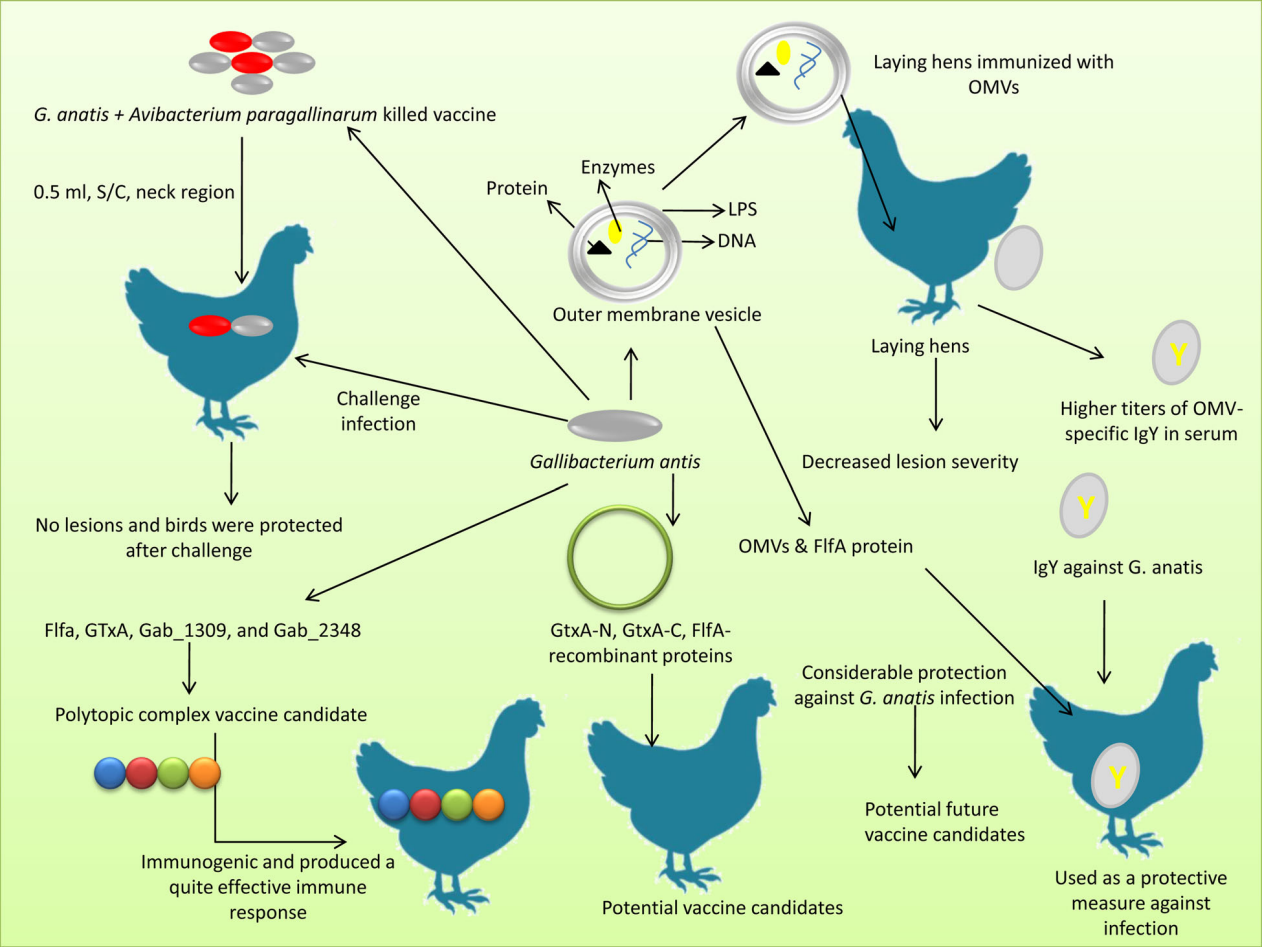
Different vaccine platforms available against *G. anatis* infection in poultry.

A polytopic complex vaccine candidate has been evaluated for vaccination potential against *G. anatis*. This *in-silico* study explored four immunogenic proteins such as Flfa, GTxA, Gab_1309, and Gab_2348 for epitope detection and prediction, and reported that these proteins are immunogenic and could generate an efficient immune response (Ataei et al. 2019). Different types of vaccines for preventing *G. anatis* infection in poultry are presented in Fig. 4.

## 9. Conclusion and future perspectives

*G. anatis* infection is an under-diagnosed or ignored cause of production loss and mortality in poultry. This opportunistic Gram-negative bacterium has hemolytic, leukocytic, and many other virulence factors that damage respiratory and reproductive tracts under disease-favoring environments such as immunosuppressive conditions and presence of co-infections. *G. anatis* causes localized as well as systemic infections that lead to rales, coughing, dyspnea, diarrhea, defective and decreased egg production, poor growth, emaciation, and death. These clinico-pathological signs are common to many other diseases of poultry, therefore proper diagnosis requires both conventional and advanced tests.

Advanced diagnostic and identification methods such as MALDI-TOF MS, FISH, qPCR, conventional PCR and sequencing can be used for rapid and precise diagnosis together with routine cultural or biochemical assays. Currently, antibiotics are the main therapeutics widely employed for treating *G. anatis* infection in poultry. However, the issue of antibiotic resistance with commonly used antibiotics including penicillin, macrolides, and tetracyclines has given rise to major concern, although this pathogen is still sensitive to novel antibiotics like ceftiofur or florfenicol. The host-pathogen interactions during co-infections need to be studied in depth to elucidate the pathogenic potentials of different *G. anatis* biovars.

Various immunogens are being explored for their vaccination potential including conventional antigens GtxA, OMVs, fimbrial antigen (FlfA), capsules, or the newly identified EF-Tu, CRISPR, and recombinant proteins (GtxA-N, GtxA-C, FlfA). Few vaccines have been developed and are being evaluated. However, for a long-term solution, proper understanding of the disease, revealing more details about the virulence factors and pathogenesis, deciphering the molecular mechanisms of pathogenicity, identifying the resistance genes in *G. anatis* that are responsible for inducing antimicrobial resistance, tackling antigenic diversity by identifying effective vaccine candidates, and exploring advanced therapeutic options are crucial for designing better prevention and control strategies against this disease to counter losses due to outbreaks of this bacterial infection.

## References

[CIT0001] Abdul-Aziz T, Fletcher OJ, Barnes HJ, Shivaprasad HL, Swayne DE. 2016. Avian histopathology. 4th ed. Florida (US): American Association of Avian Pathologists, p. 456.

[CIT0002] Addo PB, Mohan K. 1985. Atypical *Pasteurella hemolytica* type A from poultry. Avian Dis. 29(1):214–217. 3985876

[CIT0003] Aktories K, Lang AE, Schwan C, Mannherz HG. 2011. Actin as target for modification by bacterial protein toxins. FEBS J. 278(23):4526–4543. 21466657 10.1111/j.1742-4658.2011.08113.x

[CIT0004] Alispahic M, Christensen H, Hess C, Razzazi-Fazeli E, Bisgaard M, Hess M. 2011. Identification of *Gallibacterium* species by matrix-assisted laser desorption/ionization time-of-flight mass spectrometry evaluated by multilocus sequence analysis. Int J Med Microbiol. 301(6):513–522. 21596619 10.1016/j.ijmm.2011.03.001

[CIT0005] Alispahic M, Christensen H, Hess C, Razzazi-Fazeli E, Bisgaard M, Hess M. 2012. MALDI-TOF mass spectrometry confirms clonal lineages of *Gallibacterium anatis* between chicken flocks. Vet Microbiol. 160(1-2):269–273. 22728126 10.1016/j.vetmic.2012.05.032

[CIT0006] Ataei S, Bojesen AM, Amininajafi F, Ranjbar MM, Banani M, Afkhamnia M, Abtin A, Goodarzi H. 2017. First report of *Gallibacterium* isolation from layer chickens in Iran. Arch Razi Inst. 72(2):125–130.

[CIT0007] Ataei S, Ranjbar MM, Motamed N, Ataei Kachooei S, Amini Njafi F. 2019. Designing a polytopic complex vaccine candidate against *Gallibacterium anatis*: an In-silico study. Arch Razi Inst. 74(1):7–20. 31013003 10.22092/ari.2017.109804.1118

[CIT0008] Aubin GG, Haloun A, Treilhaud M, Reynaud A, Corvec S. 2013. *Gallibacterium anatis* bacteremia in a human. J Clin Microbiol. 51(11):3897–3899. 23966514 10.1128/JCM.01638-13 PMC3889794

[CIT0009] AVEC. 2011. Annual report 2011. Assoc. Poultry Processors Poultry Trade EU Countries, 41:1–52.

[CIT0010] AVEC. 2014. Association of Poultry Processors and Poultry Trade in the EU Countries-ASBL Annual Report. Rue du Luxembourg, Belgium.

[CIT0011] Bager RJ, Nesta B, Pors SE, Soriani M, Serino L, Boyce JD, Adler B, Bojesen AM. 2013. The fimbrial protein FlfA from *Gallibacterium anatis* is a virulence factor and vaccine candidate. Infect Immun. 81(6):1964–1973. 23509151 10.1128/IAI.00059-13 PMC3676021

[CIT0012] Bager RJ, Persson G, Nesta B, Soriani M, Serino L, Jeppsson M, Nielsen TK, Bojesen AM. 2013. Outer membrane vesicles reflect environmental cues in *Gallibacterium anatis*. Vet Microbiol. 167(3-4):565–572. 24090812 10.1016/j.vetmic.2013.09.005

[CIT0013] Bager RJ, Kudirkienė E, Da Piedade I, Seemann T, Nielsen TK, Pors SE, Mattsson AH, Boyce JD, Adler B, Bojesen AM. 2014. In silico prediction of *Gallibacterium anatis* pan-immunogens. Vet Res. 45(1):80–91. 25223320 10.1186/s13567-014-0080-0 PMC4423631

[CIT0014] Benson DA, Karsch‐Mizrachi I, Lipman DJ, Ostell J, Wheeler DL. 2004. GenBank: update. Nucleic Acids Res. 32:23–26. 10.1093/nar/gkh045 PMC308779 14681350

[CIT0015] Bergh PV, Heller M, Braga-Lagache S, Frey J. 2013. The *Aeromonas salmonicida* subsp. salmonicida exoproteome: global analysis, moonlighting proteins and putative antigens for vaccination against furunculosis. Proteome Sci. 11(1):44. 24127837 10.1186/1477-5956-11-44 PMC3826670

[CIT0016] Bisgaard M. 1977. Incidence of Pasteurella hemolytica in the respiratory tract of apparently healthy chickens and chickens with infectious bronchitis. Characterisation of 213 strains. Avian Pathol. 6(4):285–292. 18770338 10.1080/03079457708418238

[CIT0017] Bisgaard M, Dam A. 1981. Salpingitis in poultry. II. Prevalence, bacteriology, and possible pathogenesis in egg-laying chickens. Nord Vet Med. 33(2):81–89. 7232151

[CIT0018] Bisgaard M. 1982. Isolation and characterization of some previously unreported taxa from poultry with phenotypical characters related to *Actinobacillus*‐and *Pasteurella* species. Acta Pathol Microbiol Scand B Microbiol Immunol. 90(1‐6):59–67. 10.1111/j.1699-0463.1982.tb00081.x 7080825

[CIT0019] Bisgaard M. 1993. Ecology and significance of Pasteurellaceae in animals. Zentralbl Bakteriol B. 279(1):7–26. 10.1016/s0934-8840(11)80487-1 8369587

[CIT0020] Bisgaard M, Korczak BM, Busse HJ, Kuhnert P, Bojesen AM, Christensen H. 2009. Classification of the taxon 2 and taxon 3 complex of Bisgaard within *Gallibacterium* and description of *Gallibacterium melopsittaci* sp. nov., *Gallibacterium trehalosifermentans* sp. nov. and *Gallibacterium salpingitidis* sp. nov. Int J Syst Evol Microbiol. 59(4):735–744. 19329598 10.1099/ijs.0.005694-0

[CIT0021] Bojesen AM, Torpdahl M, Christensen H, Olsen JE, Bisgaard MJ. 2003. Genetic diversity of *Gallibacterium anatis* isolates from different chicken flocks. J Clin Microbiol. 41(6):2737–2740. 12791918 10.1128/JCM.41.6.2737-2740.2003 PMC156494

[CIT0022] Bojesen AM, Nielsen SR, Bisgaard M. 2003. Prevalence and transmission of hemolytic *Gallibacterium* species in chicken production systems with different biosecurity levels. Avian Pathol. 32(5):503–510. 14522706 10.1080/0307945031000154107

[CIT0023] Bojesen AM, Christensen H, Nielsen OL, Olsen JE, Bisgaard M. 2003. Detection of *Gallibacterium* spp. in chickens by fluorescent 16S rRNA in situ hybridization. J Clin Microbiol. 41(11):5167–5172. 14605154 10.1128/JCM.41.11.5167-5172.2003 PMC262499

[CIT0024] Bojesen AM, Nielsen OL, Christensen JP, Bisgaard M. 2004. In vivo studies of *Gallibacterium anatis* infection in chickens. Avian Pathol. 33(2):145–152. 15276980 10.1080/03079450310001652059

[CIT0025] Bojesen AM, Vazquez ME, Robles F, Gonzalez C, Soriano EV, Olsen JE, Christensen H. 2007. Specific identification of *Gallibacterium* by a PCR using primers targeting the 16S rRNA and 23S rRNA genes. Vet Microbiol. 123(1-3):262–268. 17350770 10.1016/j.vetmic.2007.02.013

[CIT0026] Bojesen AM, Vazquez ME, Gonzalez C, Aarestrup FM. 2007. Antimicrobial susceptibility of Gallibacterium from chickens in Denmark and Mexico. In World Veterinary Poultry Congress, Beijing, China.

[CIT0027] Bojesen AM, Christensen JP, Bisgaard M. 2008. Chapter 12 - *Gallibacterium* infections and other avian *Pasteurellaceae* A2 - Pattison, Mark. In: McMullin PF., Bradbury JM, Alexander DJ, editors. Poultry diseases. 6th ed. Edinburgh: W.B. Saunders; p. 160–163.

[CIT0028] Bojesen AM, Vazquez ME, Bager RJ, Ifrah D, Gonzalez C, Aarestrup FM. 2011. Antimicrobial susceptibility and tetracycline resistance determinant genotyping of *Gallibacterium anatis*. Vet Microbiol. 148(1):105–110. 20843618 10.1016/j.vetmic.2010.08.011

[CIT0029] Bojesen AM, Bager RJ, Ifrah D, Aarestrup FM. 2011. The rarely reported tet (31) tetracycline resistance determinant is common in *Gallibacterium anatis*. Vet Microbiol. 149(3-4):497–499. 21145184 10.1016/j.vetmic.2010.11.015

[CIT0030] Bojesen AM, Kristensen BM, Pors SE. 2011. The role of the capsule in the pathogenesis of *Gallibacterium anatis* in chickens. In: International Pasteurellaceae Conference (IPC), Elsinore.

[CIT0031] Bossé JT, Li Y, Sárközi R, Gottschalk M, Angen Ø, Nedbalcova K, Rycroft AN, Fodor L, Langford PR. 2017. A unique capsule locus in the newly designated *Actinobacillus pleuropneumoniae* serovar 16 and development of a diagnostic PCR assay. J Clin Microbiol. 55(3):902–907. 28053219 10.1128/JCM.02166-16 PMC5328458

[CIT0032] Bouguénec C, Bertin Y. 1999. AFA and F17 adhesins produced by pathogenic *Escherichia coli* strains in domestic animals. Vet Res. 30(2-3):318–342. 10367361

[CIT0033] Boyce JD, Adler B. 2000. The capsule is a virulence determinant in the pathogenesis of *Pasteurella multocida* M1404 (B: 2). Infect Immun. 68(6):3463–3468. 10816499 10.1128/iai.68.6.3463-3468.2000 PMC97626

[CIT0034] Carbonnelle E, Mesquita C, Bille E, Day N, Dauphin B, Beretti JL, Ferroni A, Gutmann L, Nassif X. 2011. MALDI-TOF mass spectrometry tools for bacterial identification in clinical microbiology laboratory. Clin Biochem. 44(1):104–109. 20620134 10.1016/j.clinbiochem.2010.06.017

[CIT0035] Chávez RFO, Barrios RMM, Xóchihua JAM, Chávez JFH, León JBL, Yanes MA, Martínez VAF, Mascareño JR, Escalante J. 2017. Antimicrobial resistance of *Gallibacterium anatis* isolates from breeding and laying commercial hens in Sonora. Mexico. Rev Mex Cienc Pecu. 8(3):305–312.

[CIT0036] Christensen H, Bisgaard M, Bojesen AM, Mutters R, Olsen JE. 2003a. Genetic relationships among avian isolates classified as *Pasteurella hemolytica*, ‘*Actinobacillus salpingitidis*’ or *Pasteurella anatis* with proposal of *Gallibacterium anatis* gen. nov., comb. nov.and description of additional genomospecies within *Gallibacterium* gen. nov. Int J Syst Evol Microbiol. 53(1):275–287. 12656185 10.1099/ijs.0.02330-0

[CIT0037] Christensen H, Foster G, Christensen JP, Pennycott T, Olsen JE, Bisgaard M. 2003. Phylogenetic analysis by 16S rDNA gene sequence comparison of avian taxa of Bisgaard and characterization and description of two new taxa of Pasteurellaceae. J Appl Microbiol. 95(2):354–363. 12859769 10.1046/j.1365-2672.2003.01986.x

[CIT0038] Christensen H, Kuhnert P, Busse HJ, Frederiksen WC, Bisgaard M. 2007. Proposed minimal standards for the description of genera, species and subspecies of the Pasteurellaceae. Int J Syst Evol Microbiol. 57(1):166–178. 17220461 10.1099/ijs.0.64838-0

[CIT0039] Claydon MA, Davey SN, Edwards-Jones V, Gordon DB. 1996. The rapid identification of intact microorganisms using mass spectrometry. Nat Biotechnol. 14(11):1584–1586. 9634826 10.1038/nbt1196-1584

[CIT0040] Costerton JW, Stewart PS, Greenberg EP. 1999. Bacterial biofilms: a common cause of persistent infections. Sci. 284(5418):1318–1322. 10.1126/science.284.5418.1318 10334980

[CIT0041] Dallo SF, Zhang B, Denno J, Hong S, Tsai A, Haskins W, Ye JY, Weitao T. 2012. Association of *Acinetobacter baumannii* EF-Tu with cell surface, outer membrane vesicles, and fibronectin. Sci World J. 2012:1–10. 10.1100/2012/128705 PMC3362023 22666090

[CIT0042] Dauga C. 2002. Evolution of the gyrB gene and the molecular phylogeny of Enterobacteriaceae: a model molecule for molecular systematic studies. Int J Syst Evol Microbiol. 52(2):531–547. 11931166 10.1099/00207713-52-2-531

[CIT0043] Donlan RM, Costerton JW. 2002. Biofilms: survival mechanisms of clinically relevant microorganisms. Clin Microbiol Rev. 15(2):167–193. 11932229 10.1128/CMR.15.2.167-193.2002 PMC118068

[CIT0044] El-Adawy H, Bocklisch H, Neubauer H, Hafez HM, Hotzel H. 2018. Identification, differentiation and antibiotic susceptibility of Gallibacterium isolates from diseased poultry. Ir Vet J. 71(1):5. 29441195 10.1186/s13620-018-0116-2 PMC5799919

[CIT0045] Elbestawy AR. 2014. Studies on *Gallibacterium anatis* infection in chickens (Ph.D. thesis in Poultry Diseases). Egypt: Alexandria University.

[CIT0046] Elbestawy AR, Ellakany HF, Abd El-Hamid HS, Bekheet AA, Mataried NE, Nasr SM, Amarin NM. 2018. Isolation, characterization, and antibiotic sensitivity assessment of *Gallibacterium anatis* biovar hemolytica, from diseased Egyptian chicken flocks during the years 2013 and 2015. Poult Sci. 97(5):1519–1525. 29471426 10.3382/ps/pey007

[CIT0047] El-Hamid A, Hatem S, Ellakany HF, Bekhit AA, Elbestawy AR, Elshafey MS. 2018. Effect of mixed experimental infection with *Gallibacterium anatis* and mycoplasma gallisepticum on performance of broiler chickens. AJVS. 57(1):87–97.

[CIT0048] Epstein EA, Chapman MR. 2008. Polymerizing the fibre between bacteria and host cells: the biogenesis of functional amyloid fibres. Cell Microbiol. 10 (7):1413–1420. 18373633 10.1111/j.1462-5822.2008.01148.x PMC2674401

[CIT0049] Ewers C, Janßen T, Kießling S, Philipp HC, Wieler LH. 2004. Molecular epidemiology of avian pathogenic *Escherichia coli* (APEC) isolated from colisepticemia in poultry. Vet Microbiol. 104(1-2):91–101. 15530743 10.1016/j.vetmic.2004.09.008

[CIT0050] FAOSTAT (Food and Agriculture Organization of United Nations). 2016. http://www.fao.org/faostat/en/#data.

[CIT0051] Fisher ME, Trampel DW, Griffith RW. 1998. Postmortem detection of acute septicemia in broilers. Avian Dis. 1:452–461. 9777145

[CIT0052] Frey J, Kuhnert P. 2002. RTX toxins in Pasteurellaceae. Int J Med Microbiol. 292(3-4):149–158. 12398206 10.1078/1438-4221-00200

[CIT0053] Furano AV. 1975. Content of elongation factor Tu in *Escherichia coli*. Proc Natl Acad Sci. 72(12):4780–4784. 1108000 10.1073/pnas.72.12.4780 PMC388815

[CIT0054] Garcia-Gomez E, Vaca S, Pérez-Méndez A, Ibarra-Caballero J, Pérez-Márquez V, Tenorio VR, Negrete-Abascal E. 2005. *Gallibacterium anatis*-secreted metalloproteases degrade chicken IgG. Avian Pathol. 34(5):426–429. 16236577 10.1080/03079450500267866

[CIT0055] Gellert M, Mizuuchi K, O'Dea MH, Nash HA. 1976. DNA gyrase: an enzyme that introduces superhelical turns into DNA. Proc Natl Acad Sci. 73(11):3872–3876. 186775 10.1073/pnas.73.11.3872 PMC431247

[CIT0056] Gerlach H. 1977. The significance of *Pasteurella hemolytica* in poultry. Prakt Tierartz. 58:324–328.

[CIT0057] Gilchrist P. 1963. A survey of avian respiratory diseases. Australian Vet J. 39(4):140–144.

[CIT0058] Greenham LW, Hill TJ. 1962. Observations on an avian strain of *Pasteurella hemolytica*. Vet Rec. 74:861–863.

[CIT0059] Guo LT. 2011. Studies on drug resistance and resistant genes of *Gallibacterium anatis* strains isolated from chickens in different localities. (Master Thesis in Vet. Med). China:

[CIT0060] Gürtler V, Stanisich VA. 1996. New approaches to typing and identification of bacteria using the 16S-23S rDNA spacer region. Microbiology 142(1):3–16. 8581168 10.1099/13500872-142-1-3

[CIT0061] Hacking WC, Pettit JR. 1974. Case report: *Pasteurella hemolytica* in pullets and laying hens. Avian Dis. 1:483–486. 4853549

[CIT0062] Harbourne JF. 1962. A hemolytic cocco-bacillus recovered from poultry. Vet. Rec. 74:566–567.

[CIT0063] Harper M, Boyce JD, Adler B. 2012. The key surface components of *Pasteurella multocida*: capsule and lipopolysaccharide. Curr Top Microbiol Immunol. 61:39–51. 10.1007/82_2012_202 22373812

[CIT0064] Harry EG. 1962. A hemolytic cocco-bacillus recovered from poultry. Vet Rec. 74:640.

[CIT0065] He-Ping H, Jun Z, Xia Y. 2012. Tissue distribution of *Gallibacterium anatis* in chickens co-infected with infectious bronchitis virus. J Acta Veter Zootech Sin. 43(10):1623–1629.

[CIT0066] Hess C, Grafl B, Bagheri S, Kaesbohrer A, Zloch A, Hess M. 2019. Antimicrobial resistance profiling of *Gallibacterium anatis* from layers reveals high number of multiresistant strains and substantial variability even between isolates from the same organ. Microb Drug Resist. 10.1089/mdr.2019.0056 31526229

[CIT0067] Hsieh TS, Brutlag D. 1980. ATP-dependent DNA topoisomerase from *D. melanogaster* reversibly catenates duplex DNA rings. Cell. 21(1):115–125. 6250707 10.1016/0092-8674(80)90119-1

[CIT0068] Huang WM. 1996. Bacterial diversity based on type II DNA topoisomerase genes. Annu Rev Genet. 30:79–107. 8982450 10.1146/annurev.genet.30.1.79

[CIT0069] Huangfu H, Zhao J, Yang X, Chen L, Chang H, Wang X, Li Q, Yao H, Wang C. 2012. Development and preliminary application of a quantitative PCR assay for detecting gtxA-containing *Gallibacterium* species in chickens. Avian Dis. 56(2):315–320. 22856188 10.1637/9907-082511-Reg.1

[CIT0070] Huangfu H, Xu W, Wang H, Dong Q, Guo H, Sun Y, Li Y, Gao W, Wang W, Zhang J, et al. 2018. Detection of *Gallibacterium anatis* by TaqMan fluorescent quantitative PCR. Avian Pathol. 47(3):245–252. 29243936 10.1080/03079457.2017.1416590

[CIT0071] Janetschke P, Risk G. 1970. Frequent occurrence of *Pasteurella hemolytica* in the domestic chicken in Syria. Monatsh Veterinarmed. 25(1):23–27. 5519246

[CIT0072] Johnson T, Fernandez-Alarcon C, Bojesen AM, Nolan LK, Trampel DW, Seeman T. 2011. Complete genome sequence of *Gallibacterium anatis* strain UMN179, isolated from a laying hen with peritonitis. J Bacteriol. 193 (14):3676–3677. 21602325 10.1128/JB.05177-11 PMC3133324

[CIT0073] Johnson TJ, Danzeisen JL, Trampel D, Nolan LK, Seemann T, Bager RJ, Bojesen AM. 2013. Genome analysis and phylogenetic relatedness of *Gallibacterium anatis* strains from poultry. PLoS One. 8(1):e54844. 23359626 10.1371/journal.pone.0054844 PMC3554606

[CIT0074] Jones HG, Owen DM, Kumi-Diaka J, Nagaratnam V, Rwuaan JS, Reardon MJ, Pierce KR. 1981. Reproductive tract lesions of the laying fowl with particular reference to bacterial infection. Vet Rec. 108(2):36–37. 7015681 10.1136/vr.108.2.36

[CIT0075] Jones KH, Thornton JK, Zhang Y, Mauel MJ. 2013. A 5-year retrospective report of *Gallibacterium anatis* and *Pasteurella multocida* isolates from chickens in Mississippi. Poult Sci. 92(12):3166–3171. 24235226 10.3382/ps.2013-03321

[CIT0076] Jordan FT, Williams NJ, Wattret A, Jones T. 2005. Observations on salpingitis, peritonitis and salpingoperitonitis in a layer breeder flock. Vet Rec. 157(19):573–577. 16272543 10.1136/vr.157.19.573

[CIT0077] Kjos-Hansen B. 1950. Egg peritonitis in hens caused by pathogenic cloacal bacteria. Nord Vet Med. 2:523–531.

[CIT0078] Klemm P, Schembri MA. 2000. Bacterial adhesins: function and structure. Int J Med Microbiol. 290(1):27–35. 11043979 10.1016/S1438-4221(00)80102-2

[CIT0079] Kohlert R. 1968. Studies on the etiology of inflammation of the oviduct in the hen. Monatsh Veterinarmed. 23(10):392–395. 5693194

[CIT0080] Korczak B, Christensen H, Emler S, Frey J, Kuhnert P. 2004. Phylogeny of the family Pasteurellaceae based on rpoB sequences. Int J Syst Evol Microbiol. 54(4):1393–1399. 15280320 10.1099/ijs.0.03043-0

[CIT0081] Kristensen BM, Frees D, Bojesen AM. 2010. GtxA from *Gallibacterium anatis*, a cytolytic RTX-toxin with a novel domain organisation. Vet Res. 41(3):25. 19954731 10.1051/vetres/2009073 PMC2820230

[CIT0082] Kristensen BM, Frees D, Bojesen AM. 2011. Expression and secretion of the RTX-toxin GtxA among members of the genus Gallibacterium. Vet Microbiol. 153(1-2):116–123. 21664075 10.1016/j.vetmic.2011.05.019

[CIT0083] Kudirkienė E, Bager RJ, Johnson TJ, Bojesen AM. 2014. Chaperone-usher fimbriae in a diverse selection of Gallibacterium genomes. BMC Genomics. 15(1):1093. 25495603 10.1186/1471-2164-15-1093 PMC4299563

[CIT0084] Kulp A, Kuehn MJ. 2010. Biological functions and biogenesis of secreted bacterial outer membrane vesicles. Annu Rev Microbiol. 64(1):163–184. 20825345 10.1146/annurev.micro.091208.073413 PMC3525469

[CIT0085] Lane DJ. 1991. 16S/23S rRNA sequencing. In: Stackebrandt E, Goodfellow M, editors. Nucleic acids techniques in bacterial systematics. Chichester: John Wiley & Sons, p. 115–147.

[CIT0086] Larsen P, Nielsen JL, Dueholm MS, Wetzel R, Otzen D, Nielsen PH. 2007. Amyloid adhesins are abundant in natural biofilms. Environ Microbiol. 9(12):3077–3090. 17991035 10.1111/j.1462-2920.2007.01418.x

[CIT0087] Laursen SB, Hedemand JE, Nielsen OL, Thiel S, Koch C, Jensenius JC. 1998. Serum levels, ontogeny and heritability of chicken mannan-binding lectin (MBL). Immunol. 94(4):587. 10.1046/j.1365-2567.1998.00555.x PMC1364239 9767449

[CIT0088] Lin MY, Lin KJ, Lan YC, Liaw MF, Tung MC. 2001. Pathogenicity and drug susceptibility of the Pasteurella anatis isolated in chickens in Taiwan. Avian Dis. 45(3):655–658. 11569739

[CIT0089] Lintermans PF, Bertels A, Schlicker C, Deboeck F, Charlier G, Pohl P, Norgren M, Normark S, Van Montagu M, De Greve H. 1991. Identification, characterization, and nucleotide sequence of the F17-G gene, which determines receptor binding of Escherichia coli F17 fimbriae. J Bacteriol. 173(11):3366–3373. 1675211 10.1128/jb.173.11.3366-3373.1991 PMC207947

[CIT0090] Liu LF, Liu CC, Alberts BM. 1980. Type II DNA topoisomerases: enzymes that can unknot a topologically knotted DNA molecule via a reversible double-strand break. Cell. 19(3):697–707. 6244895 10.1016/s0092-8674(80)80046-8

[CIT0091] López-Ochoa J, Montes-García JF, Vázquez C, Sánchez-Alonso P, Pérez-Márquez VM, Blackall PJ, Vaca S, Negrete-Abascal E. 2017. Gallibacterium elongation factor-Tu possesses amyloid-like protein characteristics, participates in cell adhesion, and is present in biofilms. J Microbiol. 55(9):745–752. 28865072 10.1007/s12275-017-7077-0

[CIT0092] Lucio ML, Vaca S, Vázquez C, Zenteno E, Rea I, Pérez-Márquez VM, Negrete-Abascal E. 2012. Adhesion of *Gallibacterium anatis* to chicken oropharyngeal epithelial cells and the identification of putative fimbriae. AiM. 2(04):505.

[CIT0093] MacDonald IA, Kuehn MJ. 2012. Offense and defense: microbial membrane vesicles play both ways. Res Microbiol. 163(9-10):607–618. 23123555 10.1016/j.resmic.2012.10.020 PMC3518640

[CIT0094] Majid MS, Ideris A, Aziz AR. 1986. Isolation of *Pasteurella hemolytica* from the spleen of chickens. Pertanika. 9:265–266.

[CIT0095] Mashburn-Warren LM, Whiteley M. 2006. Special delivery: vesicle trafficking in prokaryotes. Mol Microbiol. 61(4):839–846. 16879642 10.1111/j.1365-2958.2006.05272.x

[CIT0096] Mataried N. 2016. Interaction between infectious bronchitis virus and *Gallibacterium anatis* in chickens. (M.V.Sc. Thesis in Poult. Dis. Fac. Vet. Med). Egypt: Damanhour Univ.

[CIT0097] Matthes S, Löliger HC, Schubert HJ. 1969. Enzootic in chicken due to pasteurella hemolytica. Dtsch Tierarztl Wochenschr. 76(4):88–95. 5813307

[CIT0098] Matthes S, Löliger HC. 1976. Kinetics of bacterial infections in hens. Berl Munch Tierarztl Wochenschr. 89(5):98–102. 1259695

[CIT0099] Mendoza K, I, Zavaleta A, Koga Y, Rodríguez J, Alvarado A, Tinoco R. 2014. Genetic variability of *Gallibacterium anatis* strains isolated from Peruvian commercial birds with respiratory infections. Rev Investig Vet Perú. 25(2):233–244.

[CIT0100] Meneses N, Mendoza-Hernández G, Encarnación S. 2010. The extracellular proteome of Rhizobium etli CE3 in exponential and stationary growth phase. Proteome Sci. 8(1):51–61. 20942974 10.1186/1477-5956-8-51 PMC2964644

[CIT0101] Mirle C, Schöngarth M, Meinhart H, Olm U. 1991. Studies on the incidence and importance of *Pasteurella hemolytica* infections in hens, especially taking into account diseases of the laying apparatus. Monatsh Veterinarmed. 46:545–549.

[CIT0102] Miyoshi SI, Shinoda S. 2000. Microbial metalloproteases and pathogenesis. Microbes Infect. 2(1):91–98. 10717546 10.1016/s1286-4579(00)00280-x

[CIT0103] Montes-García JF, Vaca S, Vazquez-Cruz C, Soriano-Vargas E, Aguilar-Romero F, Blackall PJ, Negrete-Abascal E. 2016. Identification of a hemagglutinin from *Gallibacterium anatis*. Curr Microbiol. 72:450–456. 26729352 10.1007/s00284-015-0969-5

[CIT0104] Mráz O, Vladík P, Bohácek J. 1976. Actinobacilli in domestic fowl. Zentralbl Bakteriol Orig A. 236(2-3):294–307. 1015017

[CIT0105] Murata H, Shimada N, Yoshioka M. 2004. Current research on acute phase proteins in veterinary diagnosis: an overview. Vet J. 168(1):28–40. 15158206 10.1016/S1090-0233(03)00119-9

[CIT0106] Mushin R, Weisman Y, Singer N. 1980. *Pasteurella hemolytica* found in the respiratory tract of fowl. Avian Dis. 24 (1):162–168.

[CIT0107] Nassik S, Nassik S, Tallouzt S, Karbach N, Touzani C, Bidoudan Y, Aamarine N, Hess C. 2019. First report of isolation of Gallibacterium anatis from layer chickens in Morocco with decrease in laying performance. Avian Dis. 63(4):727–730. 31865689 10.1637/aviandiseases-D-19-00119

[CIT0108] Neubauer C, De Souza-Pilz M, Bojesen AM, Bisgaard M, Hess M. 2009. Tissue distribution of hemolytic *Gallibacterium anatis* isolates in laying birds with reproductive disorders. Avian Pathol. 38(1):1–7. 19089694 10.1080/03079450802577848

[CIT0109] Olfert ED, Godson D, Habermehl M. 1998. Endpoints in infectious disease animal models. Workshop information for pain management and human endpoint. The Johns Hopkins Center for Alternatives to Animal testing. http://altweb.jhsph.edu/science/meetings/pain/program.htm.

[CIT0110] Osuna Chávez RF, Molina Barrios RM, Hernández Chávez JF, Robles Mascareño J, Icedo Escalante JG, AcuñaYanes M. 2017. First report of biovar 6 in birds immunized against *Gallibacterium anatis* in poultry farms located in Sonora, México. Vet Mexico. 4(3):2–9.

[CIT0111] Panheleux M, Nys Y, Williams J, Gautron J, Boldicke T, Hincke MT. 2000. Extraction and quantification by ELISA of eggshell organic matrix proteins (ovocleidin-17, ovalbumin, ovotransferrin) in shell from young and old hens. Poult Sci. 79(4):580–588. 10780658 10.1093/ps/79.4.580

[CIT0112] Paudel S, Alispahic M, Liebhart D, Hess M, Hess C. 2013. Assessing pathogenicity of *Gallibacterium anatis* in a natural infection model: the respiratory and reproductive tracts of chickens are targets for bacterial colonization. Avian Pathol. 42(6):527–535. 24098932 10.1080/03079457.2013.843160

[CIT0113] Paudel S, Liebhart D, Hess M, Hess C. 2014. Pathogenesis of *Gallibacterium anatis* in a natural infection model fulfils Koch's postulates: 1. Folliculitis and drop in egg production are the predominant effects in specific pathogen free layers. Avian Pathol. 43(5):443–449. 25144260 10.1080/03079457.2014.955782

[CIT0114] Paudel S, Liebhart D, Aurich C, Hess M, Hess C. 2014. Pathogenesis of *Gallibacterium anatis* in a natural infection model fulfils Koch’s postulates: 2. Epididymitis and decreased semen quality are the predominant effects in specific pathogen free cockerels. Avian Pathol. 43(6):529–534. 25246024 10.1080/03079457.2014.967176

[CIT0115] Paudel S, Hess C, Wernsdorf P, Käser T, Meitz S, Jensen-Jarolim E, Hess M, Liebhart D. 2015. The systemic multiplication of *Gallibacterium anatis* in experimentally infected chickens is promoted by immunosuppressive drugs which have a less specific effect on the depletion of leukocytes. Vet Immunol Immunopathol. 166(1-2):22–32. 26004945 10.1016/j.vetimm.2015.05.001

[CIT0116] Paudel S, Hess M, Hess C. 2017. Coinfection of *Avibacterium paragallinarum* and *Gallibacterium anatis* in specific-pathogen-free chickens complicates clinical signs of infectious coryza, which can be prevented by vaccination. Avian Dis. 61(1):55–63. 28301236 10.1637/11481-081016-Reg

[CIT0117] Paudel S, Ruhnau D, Wernsdorf P, Liebhart D, Hess M, Hess C. 2017. Presence of *Avibacterium paragallinarum* and histopathologic lesions corresponds with clinical signs in a co-infection model with *Gallibacterium anatis*. Avian Dis. 61(3):335–340. 28957004 10.1637/11609-021317-RegR

[CIT0118] Pedersen IJ, Pors SE, Bager Skjerning RJ, Nielsen SS, Bojesen AM. 2015. Immunogenic and protective efficacy of recombinant protein GtxA-N against *Gallibacterium anatis* challenge in chickens. Avian Pathol. 44(5):386–391. 26443063 10.1080/03079457.2015.1069259

[CIT0119] Persson G, Bojesen AM. 2015. Bacterial determinants of importance in the virulence of *Gallibacterium anatis* in poultry. Vet Res. 46(1):57–67. 26063044 10.1186/s13567-015-0206-z PMC4462078

[CIT0120] Persson G, Pors SE, Thøfner ICN, Bojesen AM. 2018. Vaccination with outer membrane vesicles and the fimbrial protein FlfA offers improved protection against lesions following challenge with *Gallibacterium anatis*. Vet Microbiol. 217:104–111. 29615242 10.1016/j.vetmic.2018.03.010

[CIT0121] Pors SE, Olsen RH, Christensen JP. 2014. Variations in virulence of avian pathogenic Escherichia coli demonstrated by the use of a new in vivo infection model. Vet Microbiol. 170(3-4):368–374. 24703749 10.1016/j.vetmic.2014.02.043

[CIT0122] Pors SE, Skjerning RB, Flachs EM, Bojesen AM. 2016. Recombinant proteins from *Gallibacterium anatis* induces partial protection against heterologous challenge in egg-laying hens. Vet Res. 47(1):36–43. 26915521 10.1186/s13567-016-0320-6 PMC4766669

[CIT0123] Pors SE, Pedersen IJ, Skjerning RB, Thøfner ICN, Persson G, Bojesen AM. 2016. Outer membrane vesicles of *Gallibacterium anatis* induce protective immunity in egg-laying hens. Vet Microbiol. 195:123–127. 27771057 10.1016/j.vetmic.2016.09.021

[CIT0124] Prasai TP, Walsh KB, Bhattarai SP, Midmore DJ, Van TT, Moore RJ, Stanley D. 2017. Zeolite food supplementation reduces abundance of enterobacteria. Microbiol. Res. 195:24–30. 28024523 10.1016/j.micres.2016.11.006

[CIT0125] Proctor RA, Von Eiff C, Kahl BC, Becker K, McNamara P, Herrmann M, Peters G. 2006. Small colony variants: a pathogenic form of bacteria that facilitates persistent and recurrent infections. Nat Rev Microbiol. 4(4):295–305. 16541137 10.1038/nrmicro1384

[CIT0126] Apolinar S. R, Guerra-Inf F M, Haro-Cruz M d J. d, Miranda C. S, Morales E. M, Kristensen B.M, Bojesen A.M, Abascal E. N, Vargas E. S. 2012. Characterization of a Gallibacterium genomospecies 2 hemagglutinin. J Anim Vet Adv. 11(4):556–560.

[CIT0127] Rowe GE, Welch RA. 1994. Assays of hemolytic toxins. Meth Enzymol. 235:657–667. 10.1016/0076-6879(94)35179-1 7520121

[CIT0128] Roy K, Kjelgaard-Hansen M, Pors SE, Christensen JP, Biswas PK, Bojesen AM. 2014. Performance of a commercial Chicken-Ovo-transferrin-ELISA on the serum of brown layer chickens infected with *Gallibacterium anatis* and *Streptococcus zooepidemicus*. Avian Pathol. 43(1):57–61. 24313352 10.1080/03079457.2013.867011

[CIT0129] Rzewuska MA, Karpinska E, Szeleszczuk PI, Binek MA. 2007. Isolation of *Gallibacterium* spp. from peacocks with respiratory tract infections. Med Weter. 63(11):1431–1433.

[CIT0130] Shapiro J, Brash M, M E, Brooks A, Slavic D, McEwen B. 2013. *Gallibacterium anatis*—a review of culture-positive cases from commercial poultry submitted to the AHL in 2011 and 2012. AHL Newsletter Guelph, Ontario. Animal Health Services, Laboratory Services Division, University of Guelph, 17(1):6.

[CIT0131] Shaw DP, Cook DB, Maheswaran SK, Lindeman CJ, Halvorson DA. 1990. *Pasteurella hemolytica* as a co-pathogen in pullets and laying hens. Avian Dis. 34 (4):1005–1008. 2177972

[CIT0132] Shi Y, Tian Z, Leclercq SO, Zhang H, Yang M, Zhang Y. 2019. Genetic characterization and potential molecular dissemination mechanism of tet(31) gene in *Aeromonas caviae* from an oxytetracycline wastewater treatment system. J Environ Sci (China). 76:259–266. 30528016 10.1016/j.jes.2018.05.008

[CIT0133] Singh K, Ritchey JW, Confer AW. 2011. Mannheimia hemolytica: bacterial–host interactions in bovine pneumonia. Vet Pathol. 48(2):338–348. 20685916 10.1177/0300985810377182

[CIT0134] Singh SV, Singh BR, Sinha DK, Vinodh KO, Prasanna VA, Monika B, Sakshi D. 2016. *Gallibacterium anatis*: an emerging pathogen of poultry birds and domiciled birds. J Vet Sci Technol. 7(3):1–7.

[CIT0135] Singh SV. 2016. Studies on growth kinetics of *Gallibacterium anatis* in presence of deuterium oxide (D2O, heavy water). (M.V.Sc. Thesis submitted to Deemed University). Izatnagar, Uttar Pradesh. India: Indian Veterinary Research Institute.

[CIT0136] Singh BR, Singh SV, Palanivelu M, Kumar MA, Sinha DK, Kumar OR. 2018. *Gallibacterium anatis* outbreaks in domestic birds in North India: Antimicrobial and herbal drug sensitivity of Avibacterium and *Gallibacterium* isolates. India Journ of Poul Scien. 53(2):225–233.

[CIT0137] Soriano VE, Longinos MG, Navarrete PG, Fernández RP. 2002. Identification and characterization of *Ornithobacterium rhinotracheale* isolates from Mexico. Avian Dis. 46(3):686–690.2.0.CO;2] 12243533 10.1637/0005-2086(2002)046[0686:IACOOR]2.0.CO;2

[CIT0138] Sorour HK, Atfeehy NMA, Shalaby AG. 2015. *Gallibacterium anatis* infection in chickens and ducks. Assiut Vet Med J. 61(147):80–86.

[CIT0139] Stępień-Pyśniak D, Kosikowska U, Hauschild T, Burzyński A, Wilczyński J, Kolińska A, Nowaczek A, Marek A. 2018. A loop-mediated isothermal amplification procedure targeting the sodA gene for rapid and specific identification of *Gallibacterium anatis*. Poult Sci. 97(4):1141–1147. 29381805 10.3382/ps/pex420

[CIT0140] Stetler GL, King GJ, Huang WM. 1979. T4 DNA-delay proteins, required for specific DNA replication, form a complex that has ATP-dependent DNA topoisomerase activity. Proc Natl Acad Sci. 76(8):3737–3741. 226976 10.1073/pnas.76.8.3737 PMC383908

[CIT0141] Suh MJ, Limbach PA. 2004. Investigation of methods suitable for the matrix-assisted laser desorption/ionization mass spectrometric analysis of proteins from ribonucleoprotein complexes. Eur J Mass Spectrom (Chichester). 10(1):89–99. 15100481 10.1255/ejms.626

[CIT0142] Suzuki T, Ikeda A, Shimada J, Yanagawa Y, Nakazawa M, Sawada T. 1996. Isolation of *Actinobacillus salpingitidis*/avian *Pasteurella hemolytica*-like organisms group from diseased chickens. J Japan Vet Med Assoc. 49:800–804.

[CIT0143] Swayne DE, Glisson JR, McDougald LR, Nolan LK, Suarez DL, Nair V. 2013. Diseases of poultry.13th ed. Florida (US): Wiley Blackwell, American Association of Avian Pathologists; p. 807.

[CIT0144] Thayer SG, Beard CW. 1998. Serologic procedures. In: Swayne DE, Glisson JR, Jackwood MW, Pearson JE, Reed WM, editors. A laboratory manual for the isolation and identification of avian pathogens, 4th ed. Kenneth Square, PA: American Association of Avian Pathologists, p. 255–266.

[CIT0145] Vaca S, Monroy E, Rojas L, Vazquez C, Sanchez P, Soriano-Vargas E, Bojesen AM, Negrete-Abascal E. 2011. Adherence of *Gallibacterium anatis* to inert surfaces. J Anim Vet Adv. 10(13):1688–1693.

[CIT0146] Verbrugghe E, Boyen F, Gaastra W, Bekhuis L, Leyman B, Van Parys A, Haesebrouck F, Pasmans F. 2012. The complex interplay between stress and bacterial infections in animals. Vet Microbiol. 155(2-4):115–127. 21963418 10.1016/j.vetmic.2011.09.012

[CIT0147] Wang C, Robles F, Ramirez S, Riber AB, Bojesen AM. 2016. Culture-independent identification and quantification of *Gallibacterium anatis* (*G. anatis*) by real-time quantitative PCR. Avian Pathol. 45(5):538–544. 27171757 10.1080/03079457.2016.1184743

[CIT0148] Wang C, Pors SE, Olsen RH, Bojesen AM. 2018. Transmission and pathogenicity of *Gallibacterium anatis* and *Escherichia coli* in embryonated eggs. Vet Microbiol. 217:76–81. 29615261 10.1016/j.vetmic.2018.03.005

[CIT0149] Wang C, Pors SE, Bojesen AM. 2018. Post mortem survival of *Gallibacterium anatis* in a laying hen experimental infection model. Avian Dis. 62(2):195–200. 29613813 10.1637/11809-020818-Reg.1

[CIT0150] Willis LM, Whitfield C. 2013. Structure, biosynthesis, and function of bacterial capsular polysaccharides synthesized by ABC transporter-dependent pathways. Carbohydr Res. 378:35–44. 23746650 10.1016/j.carres.2013.05.007

[CIT0151] Wozniak RA, Fouts DE, Spagnoletti M, Colombo MM, Ceccarelli D, Garriss G, Déry C, Burrus V, Waldor MK. 2009. Comparative ICE genomics: insights into the evolution of the SXT/R391 family of ICEs. PLoS Genet. 5(12):e1000786. 20041216 10.1371/journal.pgen.1000786 PMC2791158

[CIT0152] Xie H, Huff GR, Huff WE, Balog JM, Holt P, Rath NC. 2002. Identification of ovotransferrin as an acute phase protein in chickens. Poult Sci. 81(1):112–120. 11885890 10.1093/ps/81.1.112

[CIT0153] Xie H, Newberry L, Clark FD, Huff WE, Huff GR, Balog JM, Rath NC. 2002. Changes in serum ovotransferrin levels in chickens with experimentally induced inflammation and diseases. Avian Dis. 46(1):122–131.2.0.CO;2] 11922323 10.1637/0005-2086(2002)046[0122:CISOLI]2.0.CO;2

[CIT0154] Zellner DE, Charlton BR, Cooper G, Bickford AA. 2004. Prevalence of Gallibacterium species in California poultry over 10years (1994-2003) from diagnostic specimens. In Proceedings of theAnnual Conference of the American Association of Avian Pathologists. Philadelphia, PA, USA, pp.64.

[CIT0155] Zepeda A, Ramírez S, Vega V, Morales V, Talavera M, Salgado-Miranda C, Simón-Martínez J, Bojesen AM, Soriano-Vargas E. 2009. Hemagglutinating activity of *Gallibacterium* strains. Avian Dis. 53(1):115–118. 19432013 10.1637/8375-060908-ResNote.1

[CIT0156] Zepeda VA, Calderón-Apodaca NL, Paasch ML, Martín PG, Paredes DA, Ramírez-Apolinar S, Soriano-Vargas E. 2010. Histopathologic findings in chickens experimentally infected with *Gallibacterium anatis* by nasal instillation. Avian Dis. 54(4):1306–1309. 21313855 10.1637/9423-061410-ResNote.1

[CIT0157] Zhang XP, Lu CJ, Li YT, Yang X, Wang XW, Chang HT, Liu HY, Chen L, Zhao J, Wang CQ, Chang YF. 2017. In vitro adherence and invasion of primary chicken oviduct epithelial cells by *Gallibacterium anatis*. Vet Microbiol. 203:136–142. 28619135 10.1016/j.vetmic.2017.02.009

[CIT0158] Zhang JJ, Kang TY, Kwon T, Koh H, Chandimali N, Wang XZ, Kim N, Jeong DK. 2019. Specific chicken egg yolk antibody improves the protective response against *Gallibacterium anatis* infection. Infect Immun. 87(3):e00619–18. 30559219 10.1128/IAI.00619-18 PMC6386540

